# Spatial heterogeneity dominates bacterial biogeography in the surface waters from the South China Sea by structuring environmental gradients

**DOI:** 10.1128/spectrum.00875-25

**Published:** 2025-08-12

**Authors:** Changliang Xie, Junning Gu, Linyao Peng, Chaofan Wang, Shuaishuai Xu, Shengwei Hou, Zhaohui Wang, Yali Tang

**Affiliations:** 1 College of Life Science and Technology, Jinan University 47885, Guangzhou, Guangdong, China; 2 Department of Ocean Science & Engineering, Southern University of Science and Technology 255310, Shenzhen, Guangdong, China; DePauw University, Greencastle, Indiana, USA

**Keywords:** bacterial community, biogeography, assembly, co-occurrence network, metabarcoding, environmental factors, human activity

## Abstract

Studies in environmental bacterial communities are updating our view of the powerful small-scale environmental pressures governing the bacterial community assembly. However, it remains unclear about the processes acting at broader scales, such as the thousand-kilometer scale and highly connected marine habitats. We employed a high-coverage sampling strategy combined with 16S rRNA gene sequencing to investigate the biogeography, assembly mechanisms, and co-occurrence relationships of bacterial communities in the surface waters from the intensively human-affected coasts to the open ocean of the South China Sea. Results revealed a clear geographical clustering of both environmental factors and bacterial communities from a semi-enclosed bay of the Beibu Gulf (BG), a coastal sea area of the Guangdong coast (GD), and an open region of the western South China Sea (WS). Determinant environmental drivers of bacterial communities in the three regions were also distinct: dissolved nutrients in GD, SiO_3_^2−^-Si and Chl *a* in BG, and temperature and Chl *a* in WS. The spatial and shared variations of environmental and spatial factors explained the bacterial beta-diversity dominantly. The assembly of bacterial communities was dominated by deterministic processes, particularly homogeneous selection in all regions, but showed significant regional variations in stochastic processes. Key discriminant subcommunities Pseudomonadota and Bacteroidota were assembled by notably high stochastic processes, possibly contributing to the varied bacterial assembly process. Network analysis demonstrated distinctly different co-occurrence patterns among the three regions. Our results highlight the role of spatial features associated with human activity in structuring environmental factors and then driving bacterial distribution patterns in the South China Sea.

**IMPORTANCE:**

Current research on the assembly processes of environmental bacterial communities at broader scales, such as the thousand-kilometer scale and highly connected marine habitats, remains limited. The South China Sea is the largest marginal sea in the western Pacific Ocean, exhibiting significant spatial heterogeneity across broader scales from densely human-activity coastal areas to the open ocean. Our findings reveal that the assembly mechanisms of bacterial communities along the thousand-kilometer scale from the coastal areas of the South China Sea to its western regions are dominated by homogeneous selection. Additionally, we discovered that spatial heterogeneity, by structuring local environmental gradients, plays a leading role in shaping the community structure, distribution patterns, and co-occurrence networks of surface water bacteria in the South China Sea. This study enhances the understanding of assembly mechanisms at broader scales and deepens the comprehension of the impact of spatial heterogeneity on bacterial communities.

## INTRODUCTION

Bacteria in marine surface waters are vital for maintaining marine food webs, particularly by recycling nutrients essential for phytoplankton growth, which forms the basis of the marine food chain (1, 2). They exhibit high species diversity and complex distribution patterns (3–5) and are finely regulated by multiple environmental factors, including temperature, salinity, pH, and nutrient availability (6–8).

Recently, significant progress has also been made in understanding bacterial geographical patterns (9, 10). Clear geographic clustering was observed in continuous aquatic environments for marine surface bacteria (10, 11) and sediment bacteria (12). Accompanying the geographic variation, a significant distance-decay pattern was also described as the increase in community dissimilarity with increasing spatial distance (12). All these studies proposed the importance of spatial variability to the differentiation of bacterial community (13, 14) and leave open the question of the importance of processes acting at broader scales, such as marine compartments within the same regional sea, but are biogeographically distinct (15). Generally, spatial separation tends to overwhelm the environmental effects at global scales, and environmental effects were frequently reported as the major determinant in microbial community composition at small scales (4, 10). For bacterial community differentiation at intermediate scales (10 to thousands of kilometers), the importance of environmental and spatial factors was both mentioned (4, 16), but the relative importance and the relationship of these two remain ambiguous (3, 17, 18).

Human activities significantly impact environmental gradients (19, 20), and the levels of human activity are distinct among spatial variations because of the unevenly distributed population on a larger scale (21). For example, intensive human activities in coastal areas (e.g., agricultural development, urban wastewater discharge, and marine aquaculture) create enhanced nutrient gradients (19), especially when compared to open ocean areas (22, 23). Therefore, at a larger scale, spatial features are often accompanied by differing levels of human activity and may ultimately affect bacterial distribution pattern among regions by shaping environmental gradients.

The community/subcommunity assembly mechanisms underlying bacterial distribution patterns help to understand the processes of microbial variation and maintenance and are still a hot topic today (24, 25). It is widely accepted that community/subcommunity assembly is influenced by both deterministic and stochastic processes (26, 27). Deterministic processes involve environmental filtering and biotic interactions. They include homogeneous selection (HoS), which homogenizes communities, and heterogeneous selection (HeS), which diversifies them (26, 27). Stochastic processes lead to unpredictable community changes, such as homogenizing dispersal (HD) under high dispersal rates, dispersal limitation (DL) under low dispersal rates, and drift (DR) due to random events (26). In general, environmental conditions represent ‘species sorting’ by contemporary environmental disturbances, while spatial factors represent dispersal-related processes. Spatial variation may also result in different environmental gradients, as it not only influences bacteria by varied environmental factors but also specific bacteria response to environmental gradients by potentially influencing the ecological processes that shape microbial distribution patterns (28). For instance, in areas of rapid salinity change, stochastic processes play a larger role in offshore high-salinity regions than in low-salinity estuaries (7). The interaction between bacterial taxa, which can be evaluated through co-occurrence network analysis (29), is another key aspect in understanding the bacterial community assembly. With strong interspecies interactions, bacterial communities exhibit resistance to environmental disturbances and maintain stability (30). Besides, keystone taxa, acting as the community’s core, significantly affect community structure and function (31, 32).

The South China Sea, the largest marginal sea in the western Pacific, is characterized by tropical and subtropical climates (33). The Guangdong coast (GD) is located in one of China’s most economically developed and densely populated regions, with the Pearl River system contributing large terrestrial and riverine inputs, leading to dramatic salinity variations (34, 35). The Beibu Gulf (BG) is a semi-enclosed bay in the northwestern South China Sea, where environmental and biochemical variables are heavily influenced by monsoons and Beibu Gulf currents (36, 37). In the western South China Sea (WS), drastic depth variations and a greater offshore distance result in a nutrient-poor environment (7), with continuous ocean currents and active mesoscale eddies causing rapid water exchange and disturbance (38, 39). Compared to previous localized studies on bacterial communities in the South China Sea, such as in bays (40), estuaries (7), and islands (5), our study covers a continuous spatial span of over 1,100 km, providing an ideal intermediate scale for studying the importance of spatial and environmental factors to bacterial biogeography in highly connected aquatic ecosystems from intensively human-affected coastal waters to the least affected open ocean.

Using amplicon sequencing technology, this study systematically analyzed bacterial communities in the surface waters along the coast and western regions of the South China Sea. We focused on quantifying bacterial community diversity, revealing geographical distribution patterns, elucidating assembly mechanisms, and constructing and analyzing co-occurrence network characteristics. The specific research objectives were to: (i) clarify the biogeographical patterns of the bacterial community structure and the relative importance of spatial and environmental factors to bacterial biogeography; (ii) identify the regional-specific environmental factors driven bacterial community/subcommunity; and (iii) analyze the assembly process of bacterial community, as well as the co-occurrence network under spatial heterogeneity and also with different levels of human activities.

## RESULTS

### Bacterioplankton community composition and diversity

**Fig 1:**
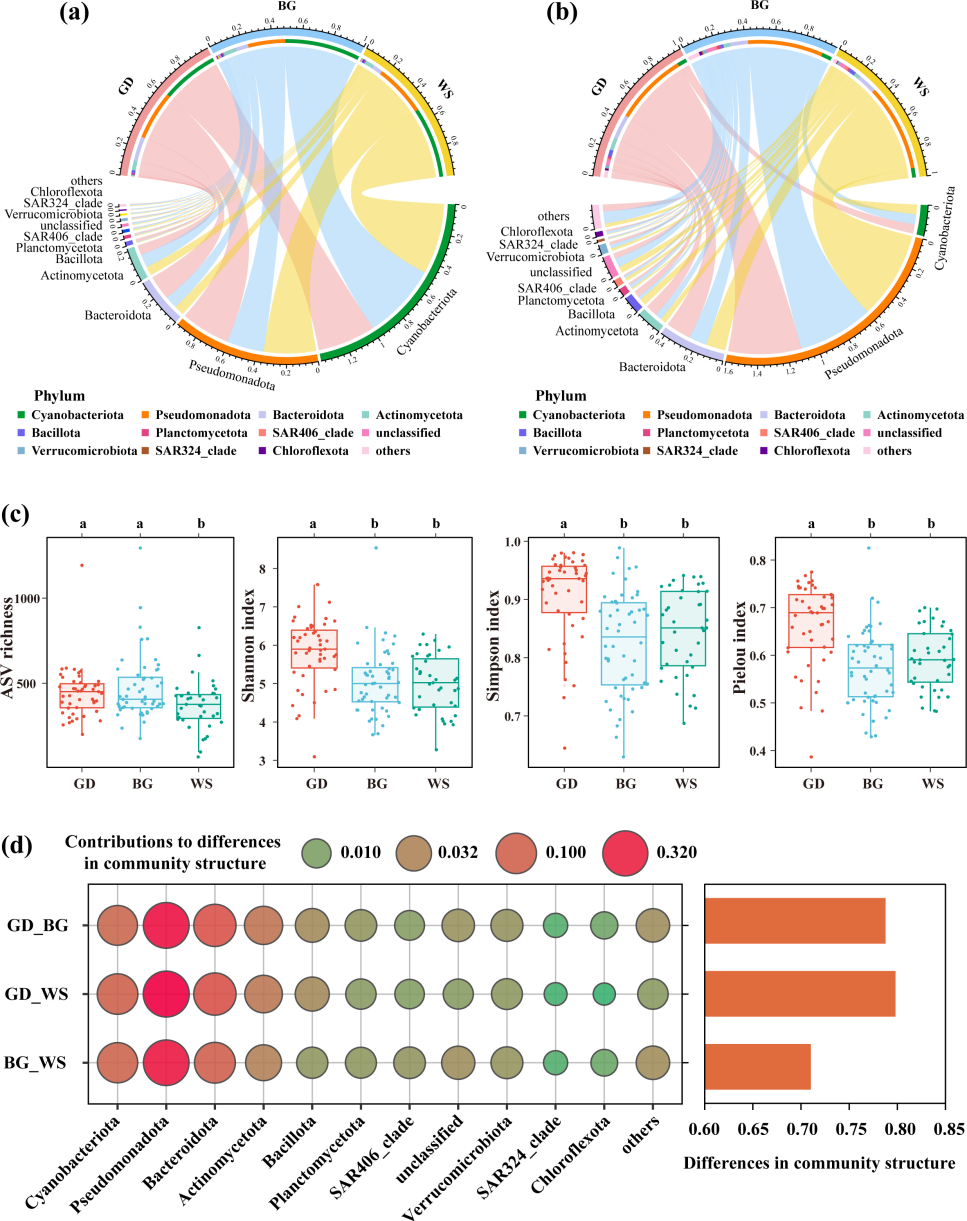
Bacterial community composition and diversity. (**a**) Taxonomic composition of bacterial communities at the phylum level showing relative abundance for the whole area and the three sea regions. The top 11 phyla by relative abundance are displayed in descending order. (**b**) Taxonomic composition at the phylum level showing relative richness for the whole area and the three sea regions. The same bacterial phyla as in (**a**) are displayed. (**c**) Bacterial community diversity in the three regions, including ASV richness, Shannon, Simpson, and Pielou indices. Different letters indicate significant differences (*P* < 0.05, Wilcoxon test). (**d**) Quantifying community structure differences based on the SIMPER analysis; balloon plots depict the contribution of the top 11 bacterial phyla in relative abundance to the community structure variability.

A total of 47 bacterial phyla, 120 classes, 263 orders, 397 families, and 909 genera were detected. Cyanobacteriota was the most dominant phylum, accounting for 46.27% of the total bacterial sequences (Fig. 1a). The proportion of Cyanobacteriota was higher in the BG compared to other regions. Bacteroidota and Actinomycetota were two other major bacterial phyla, accounting for 10.79 and 7.34%, respectively. Bacteroidota was more prevalent in the GD, while Actinomycetota had a higher relative abundance in the BG (Fig. 1a).

In terms of richness, Pseudomonadota exhibited the highest diversity with 4,119 amplicon sequence variants (ASVs), accounting for 52.55% of the total ASVs, followed by Bacteroidota (1,412 ASVs), Actinomycetota (670 ASVs), Cyanobacteriota (523 ASVs), and Bacillota (372 ASVs). Pseudomonadota dominated across the entire study area, as well as in each of the three regions and individual stations, particularly in the WS (Fig. 1b; Fig. S1). Bacteroidota and Actinomycetota followed a similar pattern, being more abundant in the GD, while the diversity of Cyanobacteriota showed no significant differences among the three regions (Fig. 1b).

Although the ASV richness in the GD was not significantly different from that in the BG (Fig. 1c), the Shannon, Simpson, and Pielou’s evenness indices were significantly higher in the GD compared to both the BG and WS, with mean values of 5.80, 0.91, and 0.66, respectively (Table S1).

The similarity percentage (SIMPER) analysis revealed that the GD and WS exhibited the largest community differences (Fig. 1d). Cyanobacteriota, Pseudomonadota, Bacteroidota, and Actinomycetota, which were the most abundant and diverse bacterial phyla, also contributed the most to community differences, accounting for approximately 10, 50, 20, and 5% of the variation between the two regions.

### Environmental factors driving bacterial community/subcommunity

**Fig 2:**
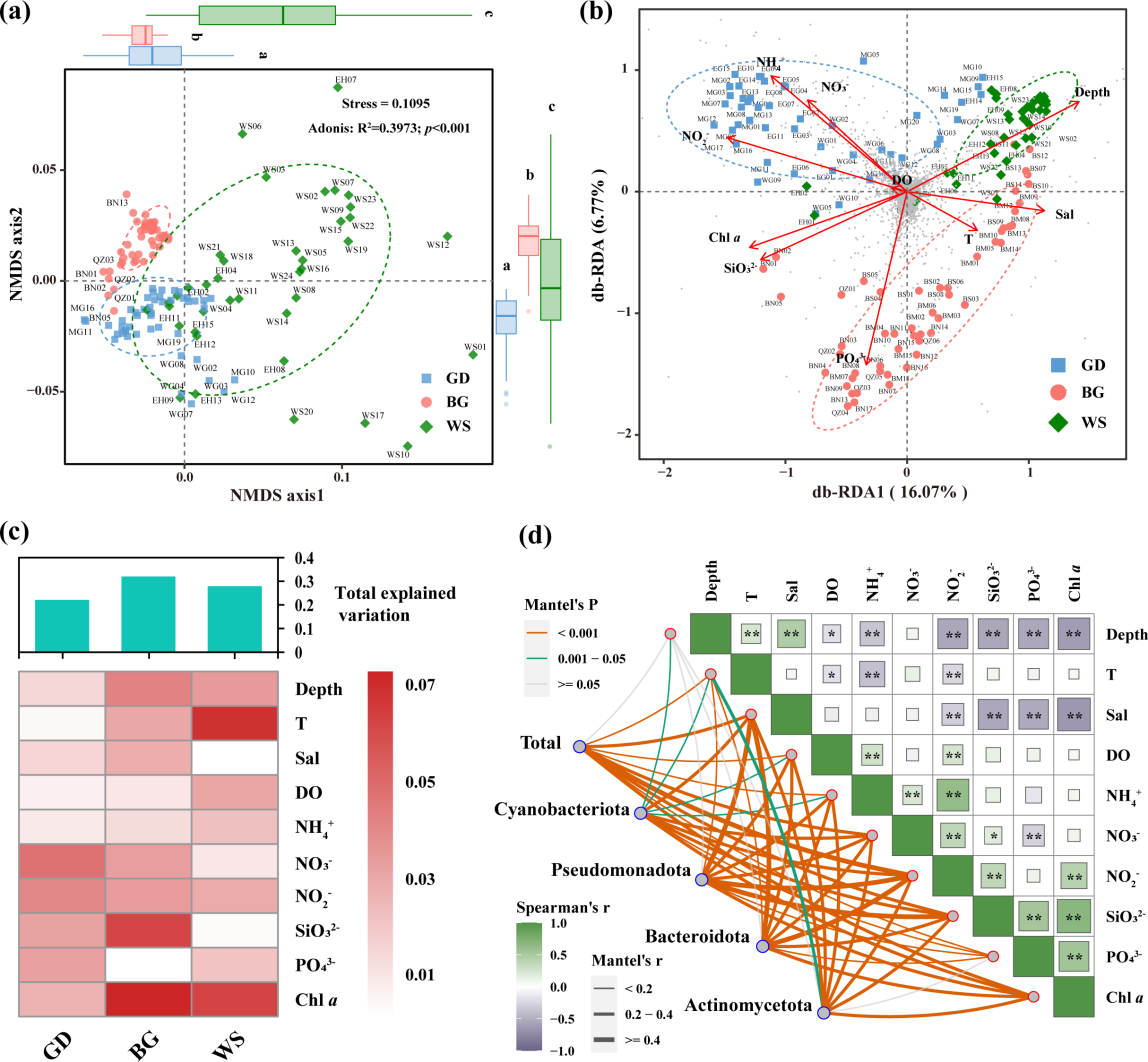
Environmental drivers of bacterial communities. (**a**) The NMDS plot showcases the variability of environmental factors across diverse marine regions. PERMANOVA was utilized to statistically assess the differences in marine environmental conditions between these regions. Furthermore, the Wilcoxon test was applied to conduct pairwise comparisons of the projections of the three marine regions onto the first and second axes of the NMDS plot. (**b**) Distance-based redundancy analysis (db-RDA) ordination plot based on Bray-Curtis distances for microbial communities. Vectors represent the direction of environmental variables; small gray points represent ASVs; and sample points are distinguished by color and shape. (**c**) Influences of the ten environmental factors on bacterial communities across the three regions using hierarchical partitioning (HP) analysis. (**d**) Environmental drivers of community/subcommunity as determined by Mantel tests. The edge width corresponds to the correlation coefficient, and the edge color indicates statistical significance. Spearman correlation coefficients between environmental factors are displayed using a color gradient. "**" denotes *P* < 0.01, and "*" denotes 0.01 ≤ *P* < .05.

The non-metric multidimensional scaling (NMDS) plot based on Euclidean distance (stress = 0.1095) suggested significant differences in environmental factors among the three regions (adonis, *P* < 0.001) (Fig. 2a; Table S2). Specifically, the GD had significantly higher concentrations of dissolved ammonium and nitrite; the BG region had significantly higher levels of dissolved phosphate; and although the WS region had lower nutrient levels, it exhibited significantly higher depth, temperature, and salinity compared to the other regions (Fig. S2). The db-RDA samples from each region typically distributed along their most prominent environmental gradients. Samples from the GD were mainly aligned with the positive direction of dissolved nitrogen compounds (NO_2_^−^-N, NO_3_^−^-N, NH_4_^+^-N), while those from the BG region were more closely associated with PO_4_^3−^-P. The WS samples clustered along the positive axes for depth and salinity.

Hierarchical partitioning (HP) analysis revealed the explanatory power of environmental factors on bacterial community variation in each region (GY: 22.13%, BG: 32.05%, WS: 27.87%) (Fig. 2c). Bacterial communities in GD were primarily influenced by dissolved nutrients, such as NO_2_^−^-N, NO_3_^−^-N, SiO_3_^2−^-Si, and PO_4_^3−^-P. In BG, SiO_3_^2−^-Si and Chl *a* were the most influential environmental factors, while temperature and Chl *a* had the greatest impact on bacterial communities in WS.

Mantel test results further revealed significant correlations between environmental variables and bacterial communities. Salinity, NO_2_^−^-N, NO_3_^−^-N, SiO_3_^2−^-Si, and Chl *a* had highly significant impacts on the overall bacterial community composition (*r* > 0.31, *P* < 0.001) (Fig. 2d). Among the subcommunities, NO_2_^−^-N was a common significant influencing factor across all four subcommunities (0.37 ≤ *r* ≤ .43, *P* < 0.001). However, cyanobacteria were the only group significantly affected by depth (*P* < 0.01). Additionally, depth and salinity at the sampling stations were significantly negatively correlated with biochemical factors, such as NO_2_^−^-N, SiO_3_^2−^-Si, PO_4_^3−^-P, and Chl *a* (Fig. 2d), suggesting that these gradients may have important indirect effects on bacterial community structure.

### Geographical pattern of bacterial community

**Fig 3:**
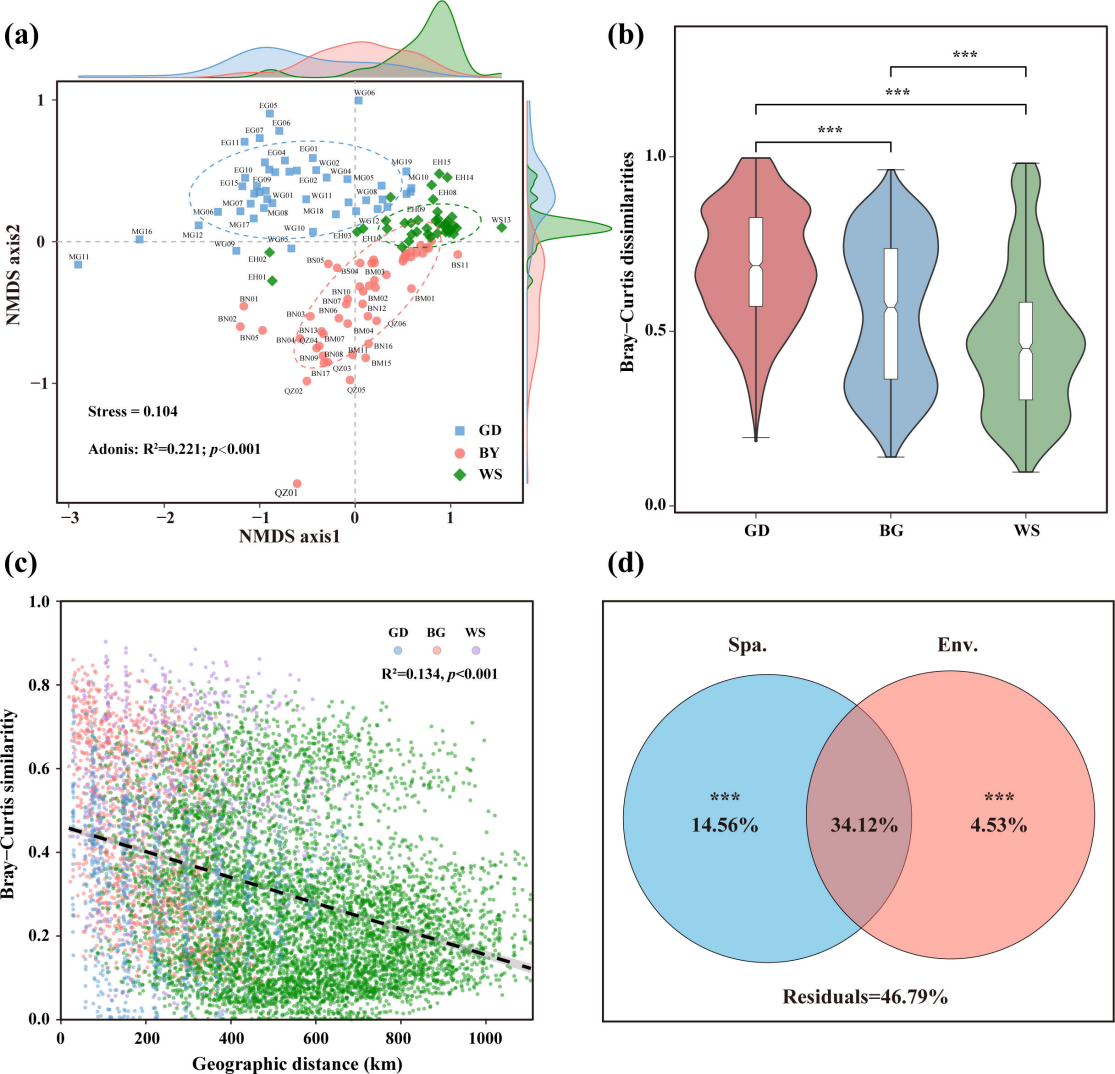
Beta diversity of bacterial communities. (**a**) NMDS plot showing community structure differences among the GD, BG, and WS regions. PERMANOVA was used to assess the significance of community differences between these regions. (**b**) Intra-regional community differences in the three regions based on Bray-Curtis distances. "***" indicates highly significant differences (*P* < 0.001, Wilcoxon test). (**c**) Linear regression between bacterial community similarity (1—Bray-Curtis distance) and geographic distance. (**d**) VP analysis of spatial and environmental factors and bacterial community. The pure effects of spatial and environmental factors were tested for significance with the permutation test (*P* < 0.001, “***”).

In the NMDS analysis based on Bray-Curtis distances, this study revealed a significant geographical separation of bacterial communities (Fig. 3a). Specifically, all collected samples were clearly clustered into three major regional groups according to their geographical locations: the GD, BG, and WS with support by permutational multivariate analysis of variance (PERMANOVA) statistical tests (*P* < 0.001) (Table S3). Further analysis using violin plots confirmed that the heterogeneity within bacterial communities varied significantly among the three regions. This intra-community heterogeneity was most pronounced in the GD, followed by the BG, and was relatively weaker in the WS, displaying a decreasing trend of GD > BG > WS (Fig. 3b).

A clear distance-decay pattern was observed in the bacterial communities, and community similarity decreased with increasing geographical distance (Fig. 3c). The geographical distance exhibited a significant negative correlation with community similarity based on the Bray-Curtis distance (*R*² = 0.134, *P* < 0.001).

The results of the variance partitioning (VP) analysis showed that spatial factors explained 48.68%; environmental factors explained 38.65%; and both factors collectively explained 53.21% of community variation (Fig. 3d).

### Assembly process of bacterial communities revealed by null model

**Fig 4:**
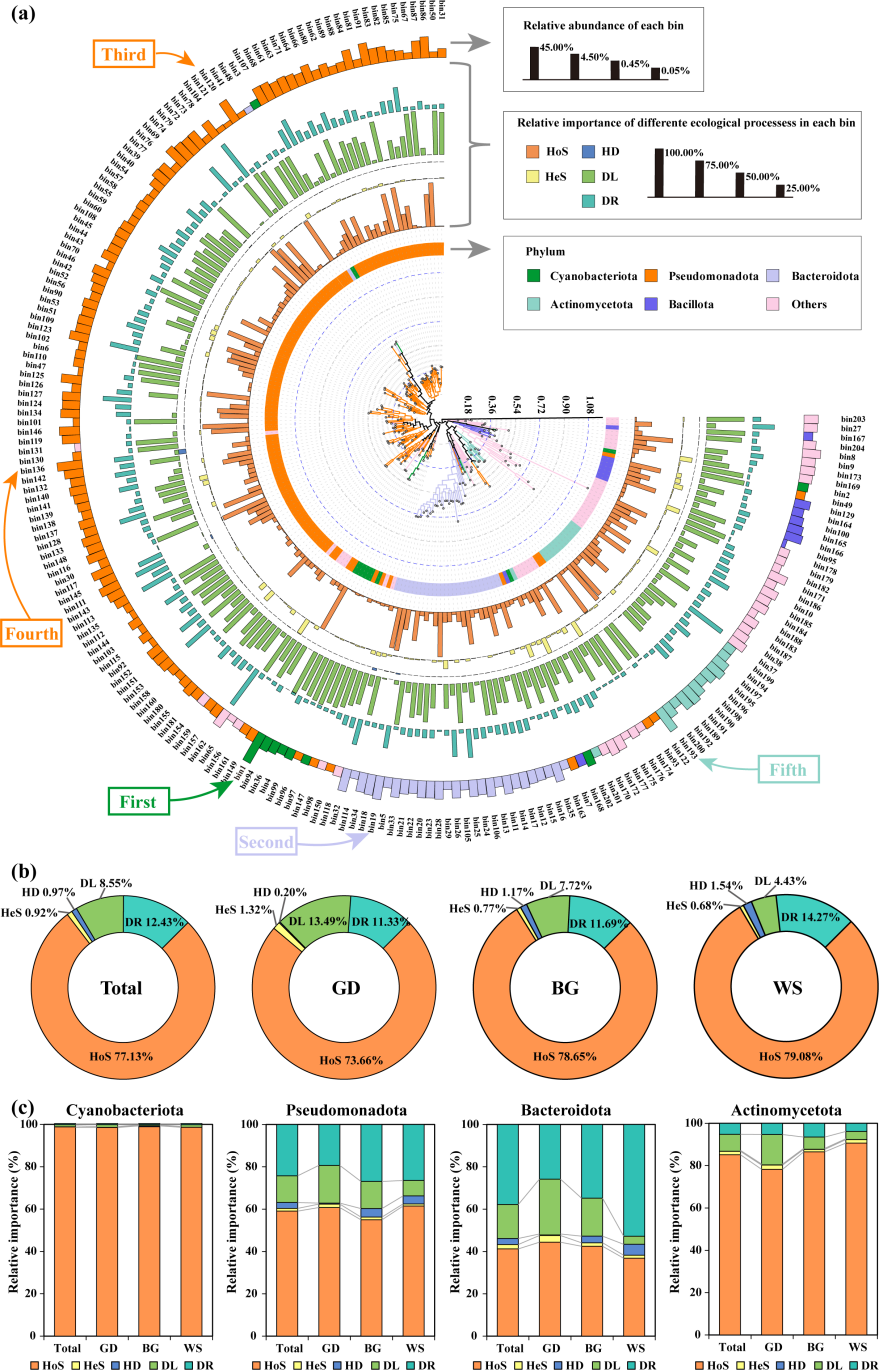
Mechanisms of the bacterial community assembly. (**a**) Assembly mechanisms of different phylogenetic bins, with the center of the circle displaying a phylogenetic tree constructed from representative ASVs within each bacterial bin. The rings, from innermost to outermost, represent the taxonomic classification at the phylum level of the representative ASVs; the contributions of homogeneous selection (HoS), heterogeneous selection (HeS), homogenizing dispersal (HD), dispersal limitation (DL), and drift (DR) to the assembly of bacterial communities in each bin are shown; the outermost ring indicates the relative abundance of each bin. Bin1, Bin19, Bin120, Bin136, and Bin193 are the five most abundant bins. (**b**) Contribution of different ecological processes to the overall region and the three specific areas. (**c**) Mechanisms of the key discriminant subcommunity assembly illustrating the assembly processes of Cyanobacteriota, Pseudomonadota, Bacteroidota, and Actinomycetota, the four bacterial phyla with the highest relative abundance and richness.

The 22,430 observed ASVs were categorized into 204 phylogenetic bins, with the assembly mechanisms for these bins shown in Fig. 4a. Overall, deterministic processes contributed far more than stochastic processes, with HoS (77.13%) being the largest contributor to the assembly, followed by DR (12.43%) and DL (8.55%). HD (0.97%) and HeS (0.92%) played smaller roles. Deterministic processes dominated in 50 bins (24.51% of bins, representing 75.18% of relative abundance), while stochastic processes dominated in 154 bins (75.49% of bins, representing 24.82% of relative abundance) (Table S4).

Across the three regions, the assembly mechanisms were similar to the overall results, with HoS being dominant in all regions (73.66–79.08%). Notably, DL contributed more to community assembly in the GD compared to the other regions (13.49%), while DR contributed more in the WS (14.27%) (Fig. 4b).

The assembly mechanisms of sub-communities from different bacterial phyla exhibited distinct variations (Fig. 4c). Overall, deterministic processes dominated the community assembly of Cyanobacteriota and Actinomycetota (>79.67%). However, Pseudomonadota and Bacteroidota showed a different pattern, with much higher stochastic processes making the main contribution (>56.69%). Assembly of Bacteroidota showed a clear stepwise decrease in dispersal limitation and an increase in DR from the GD to the WS.

### Co-occurrence network pattern of bacterial community

**Fig 5:**
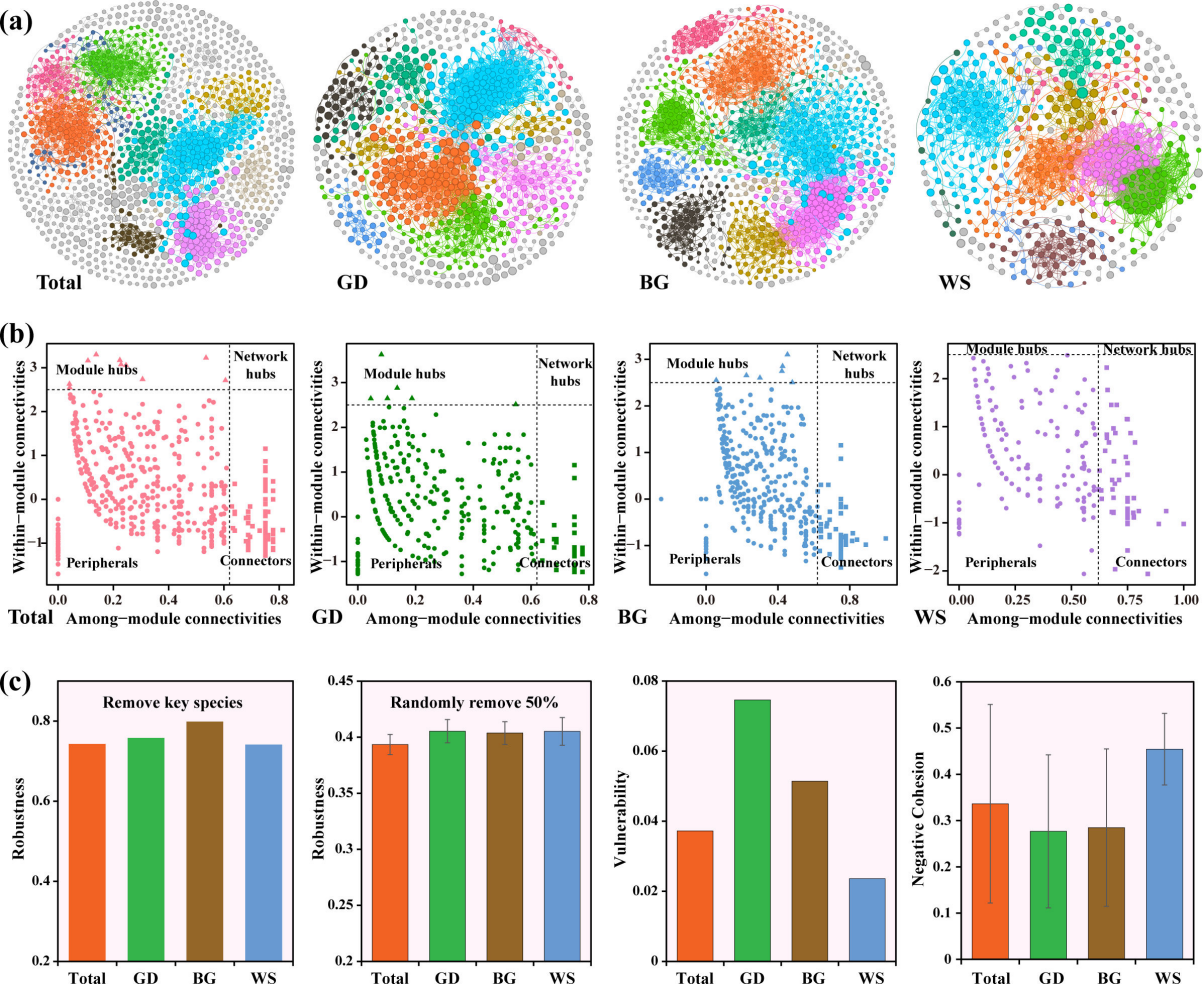
Co-occurrence network analysis. (**a**) Co-occurrence networks for the whole area and the three regions. (**b**) Key taxa identified in different regions based on their topological roles in the networks. Module hubs were identified as nodes with *Zi *≥ 2.5 and *Pi* < 0.62, connectors as nodes with *Zi *< 2.5 and *Pi* ≥ 0.62, and network hubs as nodes with *Zi *≥ 2.5 and *Pi* ≥ 0.62. (**C**) Bacterial network stability. Robustness (measured by targeted removal of key species and random removal of 50% of nodes), vulnerability (measured by the maximum node vulnerability), and negative cohesion were used to assess the network stability.

The BG exhibited a much higher proportion of negative correlations compared to the overall network and other regions (Fig. 5a, Table S5). Pseudomonadota was the dominant bacterial phylum in the networks, contributing approximately 50% (49.12–55.60%) of the network nodes, followed by Bacteroidota (17.65–23.13%) (Fig. S3 and 4).

We identified 24 module hubs and 552 connectors across the four networks. Interestingly, no network hubs were found, and no module hubs were present in the WS (Fig. 5b). Most key taxa belonged to dominant phyla identified in large modules, such as Pseudomonadota, Bacteroidota, Actinomycetota, Bacillota, and Planctomycetota.

After randomly removing 50% of the species, the robustness of the three regions was similar. Following targeted removal of key members, the BG exhibited higher resistance to the loss of key nodes (Fig. 5c). Network vulnerability (lower values indicate greater stability) demonstrated that the BG had the highest vulnerability (0.075), followed by the GD (0.051), with the WS exhibiting the lowest vulnerability (0.024) and the highest negative cohesion index (0.45 ± 0.078) (Fig. 5c), indicating the highest stability.

## DISCUSSION

By employing a high-coverage sampling strategy combined with 16S rRNA gene sequencing, we revealed the bacterial biogeography in the surface waters of the South China Sea. The study showed a clear geographical clustering of both environmental factors and bacterial communities in the intensively human-affected GD, the least affected WS, and the BG as the intermediately affected by human beings. The regional-specific environmental factors driving bacterial beta diversity, regional-specific bacteria assembly process, and regional-specific bacterial co-occurrence network were also observed. These results suggest that the spatial heterogeneity associated with different levels of human activity dominates the bacterial biogeography by shaping environmental gradients in the surface waters of the South China Sea.

A total of 47 bacterial phyla, 120 classes, 263 orders, 397 families, and 909 genera were detected. The most abundant and diverse bacterial phyla were Cyanobacteriota, Pseudomonadota, Bacteroidota, and Actinomycetota (Fig. 1a) and also contributed the most (about 10, 50, 20, and 5%, respectively) to community differences between the two regions (Fig. 1d). The dominance of Cyanobacteria is frequently detected in neighboring and global marine areas (5, 41). Human activities have provided a rich supply of nutrients that serve as substrates for the growth of heterotrophic bacteria, which in turn diminishes the competitive advantage of autotrophic cyanobacteria (42,42). The GD located near China’s most economically developed regions is closely influenced by human activities (43, 44). As a result, we observed significantly lower cyanobacteria abundance in the GD compared to the other regions. Pseudomonadota richness increases with distance from the shore (16, 45), explaining a higher relative richness of Pseudomonadota in the WS sampling stations located farther from the coast than those in other regions. Bacteroidota and Actinomycetota are two other common major bacterial phyla (5, 46) that exhibited significant regional differences among the three study areas, indicating the presence of spatial patterns in these bacterial sub-communities.

**Fig 6:**
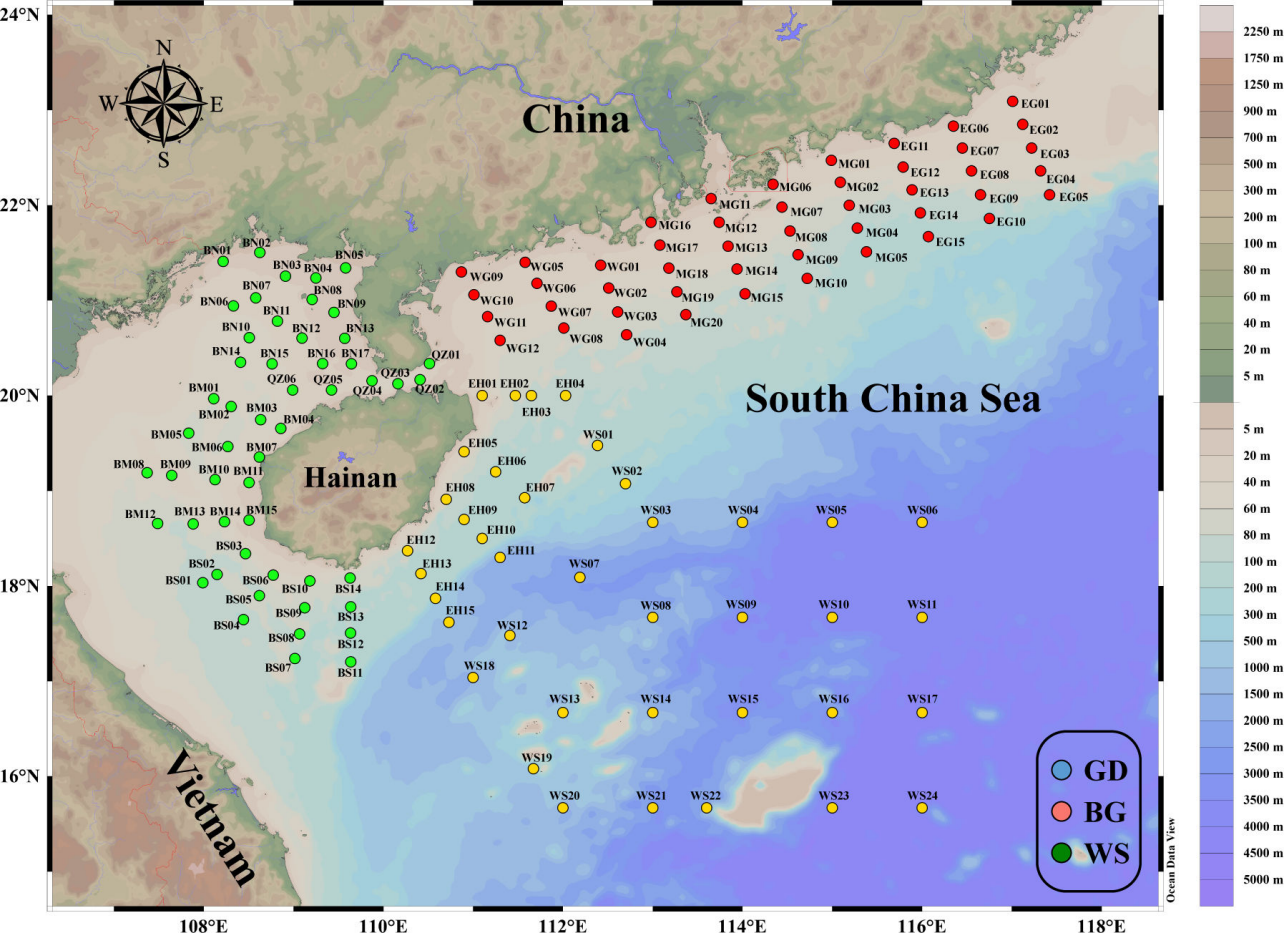
Map of the 138 sampling points distributed along the coast of the South China Sea and the western South China Sea.

Bacterial communities are often strongly influenced both directly and indirectly by major environmental factors in their habitats, such as the availability of chemical elements (7, 12, 14) and physical factors that alter living conditions (14, 47). In our study, however, the separation of the environmental factors among three regions was indicated by both NMDS and RDA analyses (Fig. 2a and b). Despite the continuum of the sampling regions, the BG, GD, and WS were located differently in longitude and latitude, showing different distances to shoreline, degree of connectivity, and shape of landscape (Fig. 6). The GD is located near the most economically developed area of China; it is intensively influenced by human activities (43, 44), and as the Pearl River system contributing large terrestrial and riverine inputs (34, 35), this region showed dramatic variations in salinity, dissolved oxygen, and nutrient concentration (Fig. S2). The WS sampling stations are located farther from the coast than those in other regions and are the most connected of the three regions (38, 39). This open ocean region has more dynamic hydrological processes with rapid water exchange processes, such as mesoscale eddies and circulation (38, 39), and is characterized by higher salinity, lower nutrient levels, and highly varied depth (Fig. S2). The BG is characterized by its three-sided land border forming a semi-enclosed region (36, 48). Nutrient diffusion in the gulf is closely related to the hydrological characteristics of the coastal waters (49) and affected by the mixing process of offshore currents (50). Hence, this region is greatly affected by land sources with the highest phosphate and large gradients. The concentration of available nutrients typically plays a significant role in promoting bacterial community diversity (40, 51). Significantly higher Shannon, Simpson, and Pielou indices were found in the nutrient-rich GD compared to other regions. Interestingly, the overall bacterial richness in the WS was significantly lower than in other regions, possibly due to its higher salinity and its coverage of more open ocean areas (51, 52).

The HP analysis further illustrated distinct determinant environmental drivers of bacterial communities among these three regions, indicating regional-specific bacterial responses to environmental factors (Fig. 2c). The bacterial communities in the GD, which is most affected by human activities, were more strongly driven by dissolved nutrients. In the semi-enclosed BG region, bacterial communities were mainly influenced by SiO_3_^2−^-Si and Chl *a*, while in the open WS region located at a lower latitude, bacterial communities were primarily driven by temperature and Chl *a*. Besides, the key discriminant subcommunity showed different associations with environmental factors as indicated by Mantel tests (Fig. 2d). Cyanobacteriota were the only group significantly affected by depth. Pseudomonadota and Bacteroidota were strongly influenced by salinity and SiO_3_^2−^-Si, and Actinomycetota was mainly influenced by salinity and NH^4+^-N. As observed in our study areas, deeper sampling sites correspond to more open and oligotrophic waters, which typically exhibit a higher dominance of Cyanobacteriota (5, 41). Salinity is crucial for bacterial communities (6), as high salinity reduces bacterial metabolic efficiency and alters nutrient cycling (53), while fluctuations in salinity can also lead to changes in the microbial community structure (7, 8). Our study highlights the significant impact of salinity variations from coastal to open waters on bacterial sub-communities (Fig. 2d).

The spatial heterogeneity may dominate the bacterial community differentiation in the surface waters of the South China Sea. This is suggested by VP analysis in our study, showing that spatial factors (14.56%) and the shared variation of environmental and spatial factors (34.12%) explained bacterial beta-diversity dominantly, much higher than environmental factors alone (4.53%) (Fig. 3d). The separation of environments and environmental drivers of bacterial communities across the three regions supports the speculations, too. Generally, at the 10 to thousand-kilometer spatial scale, environmental and spatial factors are both important to shape the microbial community composition (10). Drivers of regional bacterial community structure and diversity in thousands of kilometer scale, for example, in the Northwest Atlantic Ocean, in the entire coastal and shelf ecosystem of the China Sea in the Pacific Ocean are found to be temperature, dissolved oxygen, and salinity (8, 16, 46, 54). Several limited research studies also highlighted the unique bacterial community compositions in each of the geographical regions in highly connected marine habitats (4),proposed the importance of spatial factors (10), and suggested the biogeographic pattern of a distance-decay for marine microorganisms considering the large size and complexity of the marine ecosystem (10, 14, 16). Spatial variation structured environmental gradients controlled bacterial biogeography in the investigation of bacterial community in the coastal waters of northern Zhejiang, East China Sea (18). Our study reinforced the important role of spatial heterogeneity in structuring environment gradients and in driving bacterial community differentiation in the continuous marine habitats ranging from coastal waters to the open sea, with a distance-decay biogeographic pattern.

The fundamental mechanisms of the community/key discriminant subcommunity assembly subsequently explain the distribution of microbial communities (9, 25) and may help us understand the spatial heterogeneity-dominated bacterial geographic pattern in our study area. Across the three regions, the assembly mechanisms were similar to the overall results, with HoS being dominant in all regions (>73%) (Fig. 4b). Notably, DL contributed least to community assembly in the highly connected WS, possibly due to the low bacterial dispersal capabilities in low connective regions, as reported (11). The stochastic processes are more significant in our detected nutrient-rich GD, as when resource availability increases, the relative influence of stochastic processes also increases (55). More diverse bacterial assembly mechanisms for key discriminant sub-communities were observed. Although HoS remained the primary mechanism for sub-communities in surface waters of the South China Sea, stochastic processes dominated by DL and DR made a larger contribution in two of the most key discriminant sub-communities, Pseudomonadota and Bacteroidota, which may also explain the variations of bacterial assembly among different regions.

Spatial heterogeneity also plays a significant role in bacterial co-occurrence network stability. The WS exhibited the lowest vulnerability and highest negative cohesion index, indicating the highest stability (56–58), as supported by the network parameters of average degree and average path length (Table S5). Previous studies have suggested that environmental conditions in open ocean regions are more stable, with fewer disturbances compared to coastal areas affected by environmental gradients and human activities, supporting more stable bacterial networks (59, 60). Additionally, the movement and mixing of water masses in open ocean regions may promote microbial community dispersal and maintenance, further enhancing network stability (7, 38, 39). The BG, however, presented higher numbers of nodes, links, modules, and average clustering coefficients (Table S5), indicating more complex networks (61). Key species crucial in maintaining network structure (31, 32) also exhibited significant spatial heterogeneity. Despite highest stability, bacteria in the WS were the most dependent on keystone species (also key discriminant subcommunity Pseudomonadota) for maintenance, as suggested by the robustness analysis.

In this study, we discussed the spatial characteristics of large-scale marine bacterial communities by sampling only once and mainly in summer. However, many important natural phenomena, such as seasonal variations, are important for bacterial communities' assemblage (39, 62), which were not investigated in this study and need further exploration.

### Conclusion

By employing a high-coverage sampling strategy combined with 16S rRNA gene sequencing to investigate the biogeography, assembly mechanisms, and co-occurrence relationships of bacterial communities in the surface waters along the thousand-kilometer scale of the South China Sea, the study reveals the bacterial biogeography in this area and found the crucial role of spatial heterogeneity accompanied with human activity in structuring. The environmental gradients affect the bacterial assembly process and co-occurrence network and thus shape the bacterial biogeographical pattern.

## MATERIALS AND METHODS

### Sample collection and environmental attribute analysis

The South China Sea (longitude 105°–118°E, latitude 4°–21°N) located in the western Pacific Ocean is one of China’s three major marginal seas. Samples were collected mainly in summer between June and August 2020 and August and September 2021. Samples were characterized as the GD, WS, and BG according to their geographic features of the sampling sites. The GD included sampling sites within the range of China’s Guangdong Province coastline to the South China Sea continental shelf; the BG included sampling sites within the Beibu Gulf and at its mouth; and the WS included sampling sites in the waters east of Hainan Province, the Xisha Islands, and the surrounding deep sea areas. These three regions share different hydrological conditions. The GD receives large input of fresh water from the Pearl River (63) and forms an along-coast circulation and an upwelling driven by the East Asian monsoon in summer (64). The BG shows cyclonic circulation within the bay also in summer (36). The WS is mainly affected by the west bundary current of the South China Sea and the cyclonic circulation of the South China Sea highly connected to the open ocean (65, 66). These samples were obtained during research voyages on the ships "Shiyan 2," "Shiyan 3," "Yuezhan Yuke 9," and "Haike 68" as part of the National Natural Science Foundation of China shared voyages NORC2020-07 and NORC2021-11. Our sampling range spanned more than 1,100 km, yielding a total of 138 samples, and was divided into three major regions based on geographical location: the GD (47 samples), the WS (39 samples), and the BG (52 samples) (Fig. 6).

We used a submersible pump to collect 1–4 L of seawater from a depth of 2.0 m for the bacterial 16S amplicon analysis. Seawater was filtered through 0.2 µm polycarbonate membranes (Millipore, USA), and the filters were promptly stored at −80°C until DNA extraction. A conductivity-temperature-depth recorder (CTD SBE-911 plus, Sea Bird Electronics, Inc., USA), a multiparameter sonde YSI 6600, and an oxygen SBE 43 sensor were used to measure temperature, depth (referring to the water depth at the sampling stations), salinity, chlorophyll *a* (Chl *a*), pH, and dissolved oxygen (DO) *in situ* at each sampling site. Dissolved inorganic nutrients, including DIN (NO_2_^−^-N, NO_3_^−^-N, NH_4_^+^-N), DIP (PO_4_^3−^-P), and DSi (SiO_3_^2−^-Si), were measured in triplicate via the spectrophotometric method (67) with detection limits of 0.02, 0.05, 0.02, 0.01, and 0.02 µmol/L, respectively, for NO_2_^−^, NO_3_^−^, NH_4_^+^, PO_4_^3−^, and SiO_3_^2−^.

### DNA extraction, PCR amplification, and sequencing

Total DNA was extracted from the filters using Qiagen’s PowerSoil DNA Isolation Kit (Qiagen, Germany) following the manufacturer’s instructions. The V3–V4 regions of the bacterial 16S rRNA gene were amplified by PCR using the forward primer 338F and reverse primer 806R (68). The 20 µL PCR reaction mixture contained 4 µL 5× TransStart FastPfu buffer, 2 µL 2.5 mM dNTPs, 0.8 µL forward primer, 0.8 µL reverse primer, 0.4 µL TransStart FastPfu DNA polymerase, and 10 ng template DNA. The PCR (T100 Thermal Cycler, Bio-Rad, USA) amplification program was as follows: initial denaturation at 95°C for 3 min, followed by 27 cycles of 95°C for 30 s, 55°C for 30 s, 72°C for 30 s, and a final extension at 72°C for 10 min, then held at 4°C. The amplicons were purified using an AxyPrep DNA Gel Extraction Kit (Axygen Biosciences, USA) and quantified with a Qubit 4.0 fluorometer (Thermo Fisher Scientific, USA). The purified PCR products were then sequenced on an Illumina PE250 platform (Shanghai Majorbio Bio-Pharm Technology Co., Ltd., China) using the NEXTFLEX Rapid DNA-Seq Kit.

### Sequencing data processing

Paired-end raw sequences were quality-controlled using fastp software (https://github.com/OpenGene/fastp, version 0.19.6) (69) and merged using FLASH (http://www.cbcb.umd.edu/software/flash, version 1.2.11) (70). The quality-controlled merged sequences were denoised and chimera-checked using the DADA2 plugin in Qiime2 (71), generating amplicon sequence variants (ASVs). Sequences annotated as chloroplasts or mitochondria were removed from all samples. Taxonomic assignment of ASVs was performed using a naive Bayes classifier trained on the Silva 16S rRNA gene database (v138.1) in Qiime2.

### Statistical analysis

Alpha diversity indices for each sample and beta diversity indices (Bray-Curtis dissimilarity) between sample pairs were calculated using the "vegan" package (version 2.6-4) in R software (version 4.1.0). Wilcoxon tests were performed using the "ggpubr" package (version 0.6.0) to compare alpha and beta diversities between regions (8). The similarity percentage (SIMPER) analysis was conducted using the "simper" function to quantify community differences (72). Non-metric multidimensional scaling (NMDS) was conducted using "vegan" and "ggplot2" (version 3.4.4), and group differences were tested with permutational multivariate analysis of variance (PERMANOVA) using the "adonis" function in "vegan" (73). Geographical distances between sampling points were calculated using longitude and latitude data with the "geosphere" package (version 1.5-18). To assess the influence of environmental variables on the bacterial community structure, distance-based redundancy analysis (db-RDA) and Mantel tests were performed using the "capscale" function in "vegan" and the "mantel test" function in the "linkET" package (version 0.0.7.4). The Cartesian coordinate analysis was conducted with the "SoDA" package (version 1.0-6), and dbMEM was executed with the "adespatial" package (version 0.3-21). Variation partitioning (VP) and hierarchical partitioning (HP) were performed using the "rdacca.hp" package (version 1.1-0) to calculate the explanatory power of spatial and environmental factors on community variation (74).

To quantify the assembly mechanisms of bacterial communities and sub-communities, we used the iCAMP package (version 1.5.12) and a phylogenetic bin-based null model (75). A phylogenetic signal threshold of 0.2 was set, and the minimum number of bins was set at 36. The contribution of each process to the whole community was calculated by weighting the score of each bin by its relative abundance. The icamp.cate function was used to assess the relative importance of each process for subcommunities (25, 75).

Co-occurrence networks were constructed using Spearman correlation to visualize ASV interactions. Only ASVs present in three or more samples were included in the analysis. The "Hmisc" package (version 5.0-1) was used to compute Spearman correlation coefficients (|*r*| > 0.7), and *P*-values (*P* < 0.01) were adjusted using the Benjamini-Hochberg method (BH) (Harrell FE Jr, Harrell MFE Jr, Package ‘hmisc’. CRAN, 2019). Network visualization and topological analyses were performed using Gephi software (version 0.10.0) (73). To identify keystone taxa, the "ggClusterNet" package (version 0.1.0) was used to calculate within-module (*Zi*) and between-module (*Pi*) connectivities (56). The "ggClusterNet" package was also used to assess network stability, including robustness to targeted and random node removal as well as vulnerability. Negative cohesion, an additional measure of stability, was calculated as per methods provided by Yuan et al. (58).

Figure 6 was generated usding the visualization software ODV (Ocean Data View, Schlitzer, Reiner, odv.awi.de, 2025.)

## Data Availability

The raw sequencing data have been deposited in the NCBI Short Read Archive database under the BioProject code PRJNA1172150.

## SUPPLEMENTAL MATERIAL

Supplemental material spectrum.00875-25-s0001.docx Fig. S1 to S4; Tables S1 to S6.

ASM does not own the copyrights to Supplemental Material that may be linked to, or accessed through, an article. The authors have granted ASM a non-exclusive, world-wide license to publish the Supplemental Material files. Please contact the corresponding author directly for reuse.
