# Supplementary material for: Spatial heterogeneity dominates bacterial biogeography in the surface waters from the South China Sea by structuring environmental gradients
Source: Microbiol Spectr. 2025 Aug 12;13(9):e00875-25. doi: 10.1128/spectrum.00875-25 (PMC12403860; doi:10.1128/spectrum.00875-25)
Supplement: Supplemental material — Fig. S1 to S4; Tables S1 to S6. [file spectrum.00875-25-s0001.docx]

**Supplementary Materials**


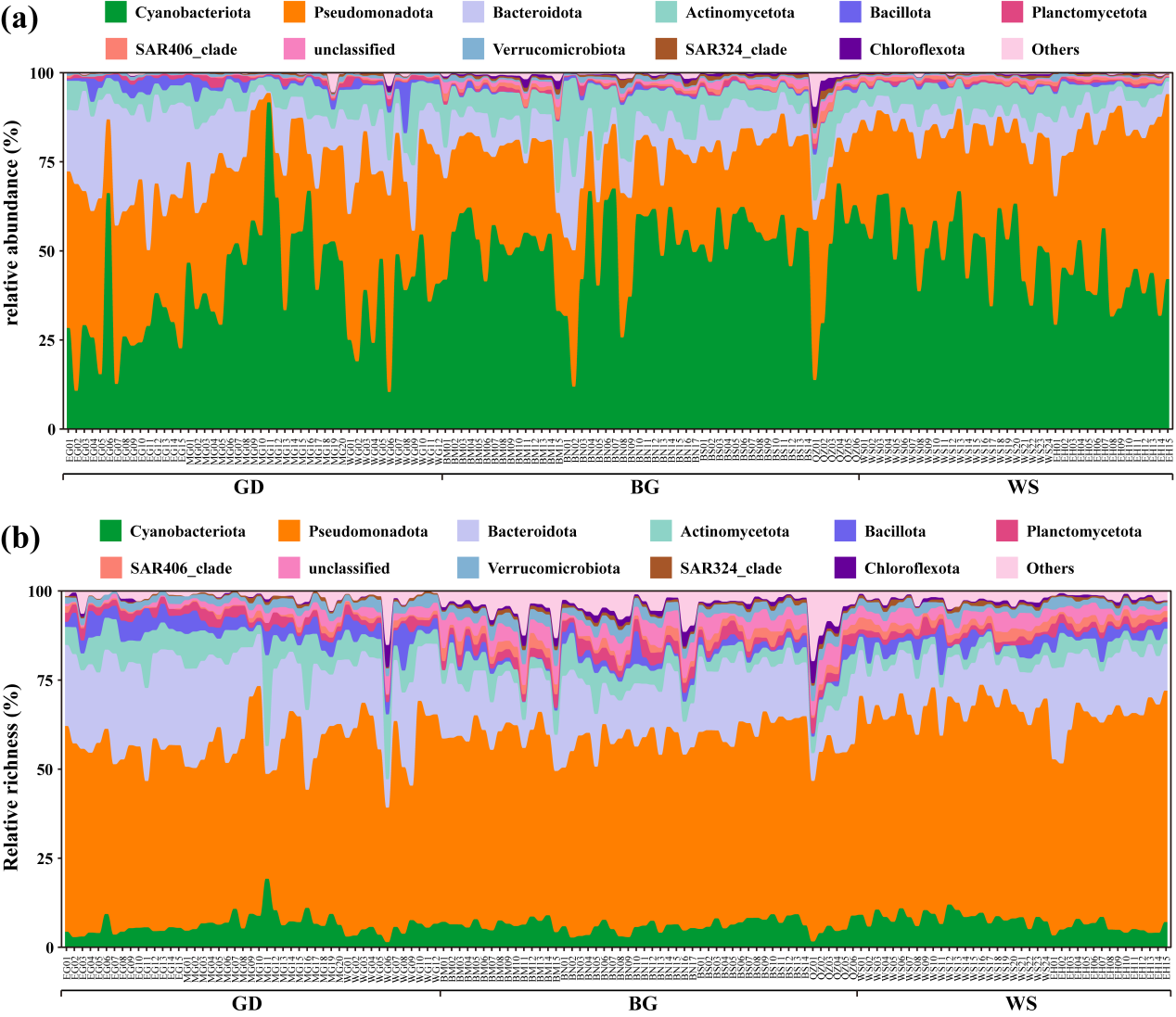


Fig. S1 Compositional variations in bacterial abundance (a) and richness (b) at the phylum level for each sampling site. The figure depicts the top 11 bacterial phyla based on their abundance percentage.


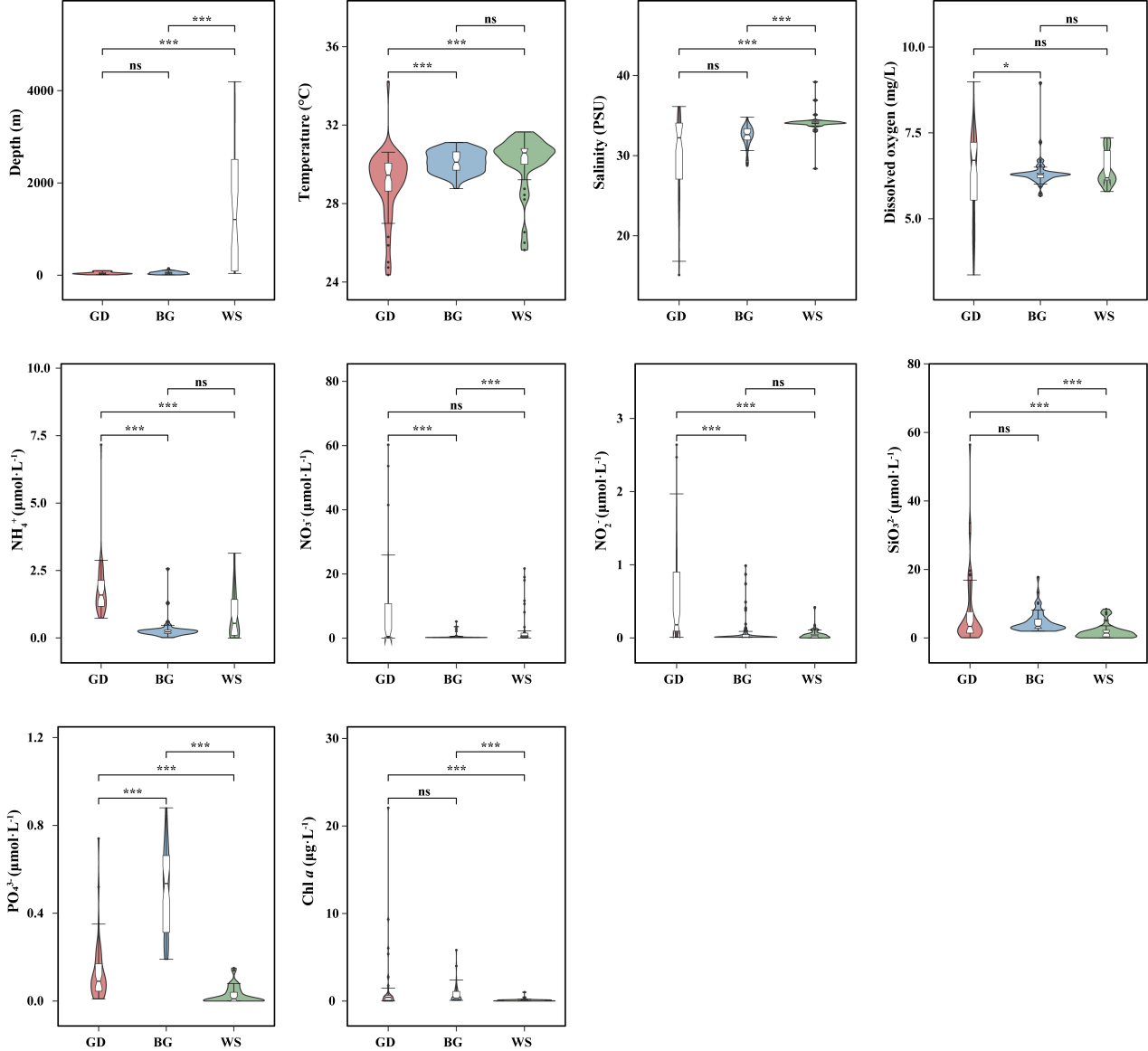


Fig. S2 Violin plots illustrate the variation of 10 environments in the three seas. Multiple comparisons of each environmental factor were performed using the Wilcoxon test, *p*<0.001, “***”; 0.001≤*p*<0.01, “**”; 0.01≤*p*<0.05, “*”; *p*≥0.05, “ns”.


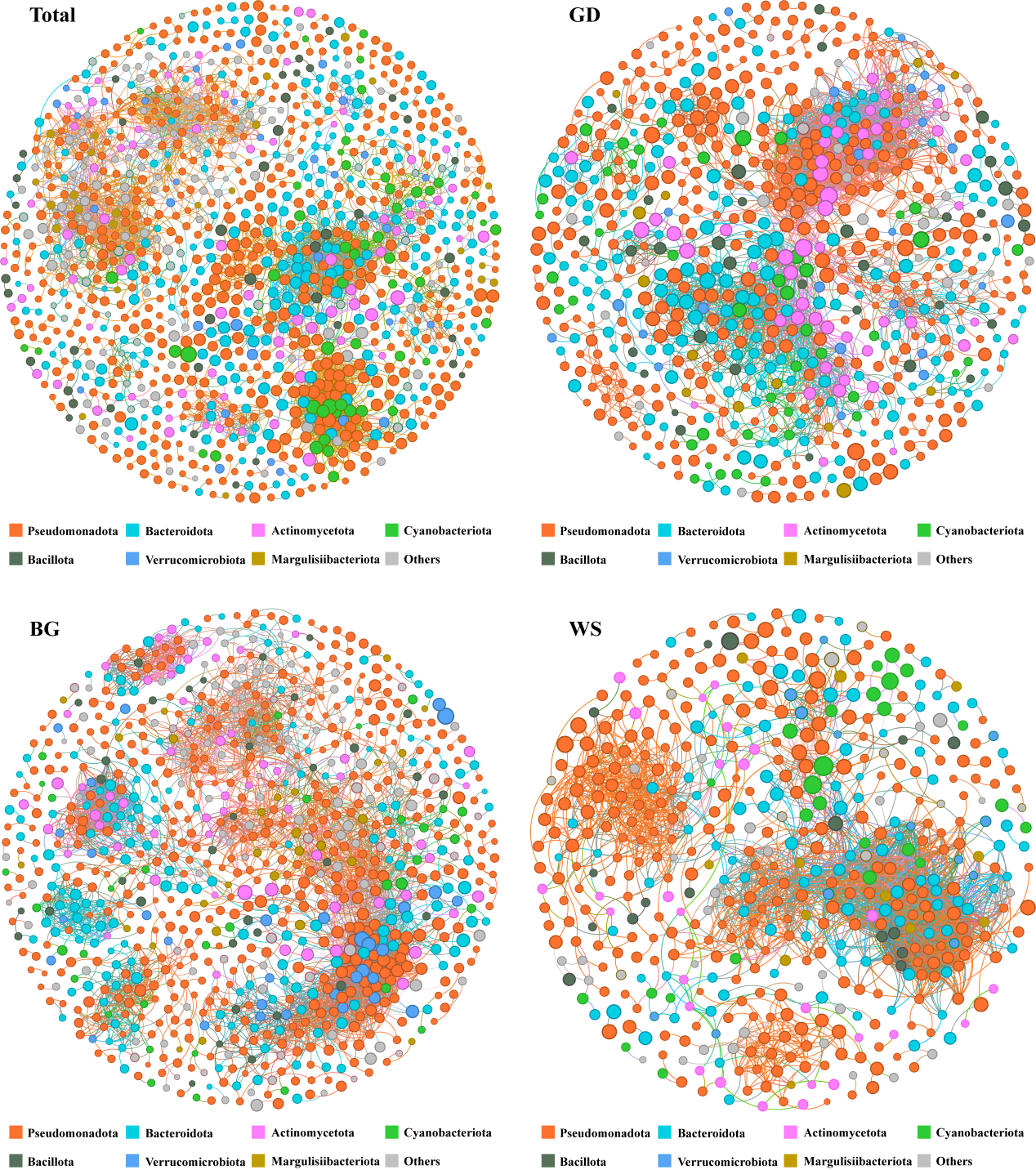


Fig. S3 Bacterial co-occurrence network colored by different phyla. In each network, the 7 phyla with the highest richness are assigned different colors for distinction, while bacteria belonging to other phyla are categorized as "Others". Node sizes correspond to the relative abundance of DNA reads.


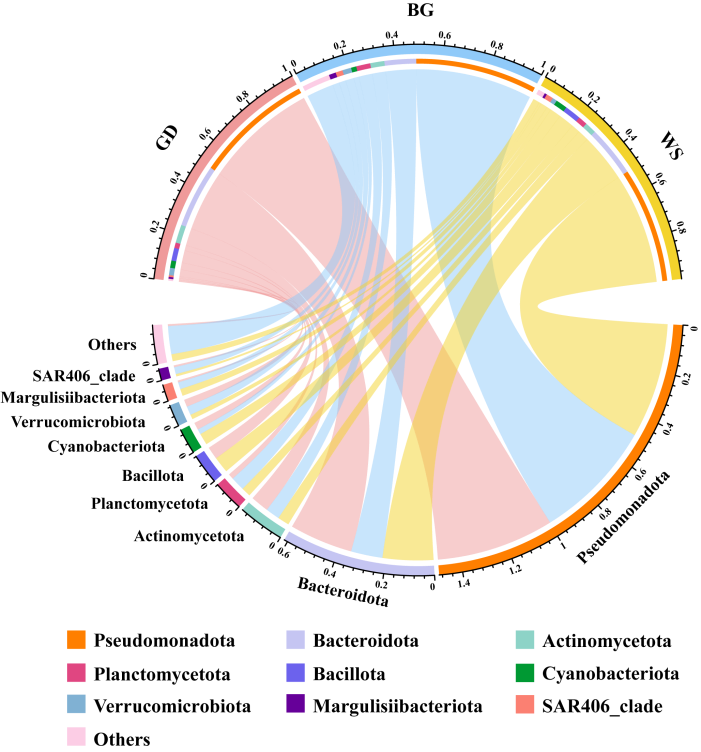


Fig. S4 Community composition and distribution of key species in the network across the three sea areas.

Table S1 The α diversity of all sampling sites.

| **Sea area** | **Station** | **Richness** | **Shannon** | **Simpson** | **Pielou** |
| --- | --- | --- | --- | --- | --- |
| GD | EG01 | 277 | 5.90 | 0.94 | 0.73 |
| GD | EG02 | 423 | 6.52 | 0.97 | 0.75 |
| GD | EG03 | 467 | 6.11 | 0.93 | 0.69 |
| GD | EG04 | 535 | 6.70 | 0.96 | 0.74 |
| GD | EG05 | 450 | 6.64 | 0.98 | 0.75 |
| GD | EG06 | 279 | 4.43 | 0.76 | 0.55 |
| GD | EG07 | 479 | 6.73 | 0.97 | 0.76 |
| GD | EG08 | 503 | 6.47 | 0.96 | 0.72 |
| GD | EG09 | 561 | 7.01 | 0.98 | 0.77 |
| GD | EG10 | 584 | 7.12 | 0.98 | 0.77 |
| GD | EG11 | 268 | 5.87 | 0.94 | 0.73 |
| GD | EG12 | 464 | 6.11 | 0.95 | 0.69 |
| GD | EG13 | 305 | 6.13 | 0.95 | 0.74 |
| GD | EG14 | 356 | 6.31 | 0.95 | 0.74 |
| GD | EG15 | 404 | 6.55 | 0.97 | 0.76 |
| GD | MG01 | 389 | 5.30 | 0.90 | 0.62 |
| GD | MG02 | 491 | 6.63 | 0.97 | 0.74 |
| GD | MG03 | 479 | 6.43 | 0.96 | 0.72 |
| GD | MG04 | 465 | 6.35 | 0.96 | 0.72 |
| GD | MG05 | 552 | 6.50 | 0.96 | 0.71 |
| GD | MG06 | 319 | 5.37 | 0.88 | 0.65 |
| GD | MG07 | 427 | 5.76 | 0.92 | 0.66 |
| GD | MG08 | 455 | 5.92 | 0.93 | 0.67 |
| GD | MG09 | 405 | 4.80 | 0.81 | 0.55 |
| GD | MG10 | 397 | 4.50 | 0.79 | 0.52 |
| GD | MG11 | 255 | 3.09 | 0.64 | 0.39 |
| GD | MG12 | 298 | 4.85 | 0.86 | 0.59 |
| GD | MG13 | 487 | 6.25 | 0.95 | 0.70 |
| GD | MG14 | 510 | 5.45 | 0.88 | 0.61 |
| GD | MG15 | 511 | 5.20 | 0.84 | 0.58 |
| GD | MG16 | 326 | 4.09 | 0.73 | 0.49 |
| GD | MG17 | 441 | 6.20 | 0.94 | 0.71 |
| GD | MG18 | 453 | 4.82 | 0.81 | 0.55 |
| GD | MG19 | 588 | 5.58 | 0.87 | 0.61 |
| GD | MG20 | 573 | 5.79 | 0.91 | 0.63 |
| GD | WG01 | 468 | 6.20 | 0.95 | 0.70 |
| GD | WG02 | 335 | 5.86 | 0.94 | 0.70 |
| GD | WG03 | 379 | 6.01 | 0.94 | 0.70 |
| GD | WG04 | 386 | 5.71 | 0.94 | 0.66 |
| GD | WG05 | 200 | 4.72 | 0.82 | 0.62 |
| GD | WG06 | 1192 | 7.58 | 0.98 | 0.74 |
| GD | WG07 | 542 | 5.76 | 0.89 | 0.63 |
| **Sea area** | **Station** | **Richness** | **Shannon** | **Simpson** | **Pielou** |
| GD | WG08 | 582 | 6.28 | 0.94 | 0.68 |
| GD | WG09 | 355 | 5.63 | 0.92 | 0.66 |
| GD | WG10 | 392 | 4.16 | 0.75 | 0.48 |
| GD | WG11 | 291 | 5.66 | 0.93 | 0.69 |
| GD | WG12 | 485 | 5.80 | 0.91 | 0.65 |
| **Average** | | **442.19** | **5.80** | **0.91** | **0.66** |
| BG | BM01 | 548 | 5.82 | 0.91 | 0.64 |
| BG | BM02 | 483 | 4.65 | 0.76 | 0.52 |
| BG | BM03 | 578 | 4.63 | 0.75 | 0.50 |
| BG | BM04 | 272 | 4.51 | 0.76 | 0.56 |
| BG | BM05 | 318 | 5.23 | 0.88 | 0.63 |
| BG | BM06 | 502 | 5.85 | 0.91 | 0.65 |
| BG | BM07 | 464 | 4.55 | 0.77 | 0.51 |
| BG | BM08 | 386 | 5.27 | 0.88 | 0.61 |
| BG | BM09 | 379 | 5.32 | 0.88 | 0.62 |
| BG | BM10 | 236 | 5.13 | 0.88 | 0.65 |
| BG | BM11 | 583 | 4.73 | 0.79 | 0.51 |
| BG | BM12 | 313 | 5.19 | 0.89 | 0.63 |
| BG | BM13 | 324 | 5.39 | 0.90 | 0.65 |
| BG | BM14 | 379 | 5.22 | 0.87 | 0.61 |
| BG | BM15 | 944 | 6.14 | 0.91 | 0.62 |
| BG | BN01 | 357 | 6.03 | 0.96 | 0.71 |
| BG | BN02 | 407 | 6.24 | 0.97 | 0.72 |
| BG | BN03 | 636 | 5.78 | 0.87 | 0.62 |
| BG | BN04 | 379 | 4.07 | 0.73 | 0.48 |
| BG | BN05 | 582 | 6.06 | 0.93 | 0.66 |
| BG | BN06 | 364 | 3.66 | 0.66 | 0.43 |
| BG | BN07 | 393 | 3.70 | 0.63 | 0.43 |
| BG | BN08 | 758 | 6.32 | 0.95 | 0.66 |
| BG | BN09 | 492 | 5.52 | 0.90 | 0.62 |
| BG | BN10 | 405 | 4.36 | 0.72 | 0.50 |
| BG | BN11 | 380 | 4.29 | 0.71 | 0.50 |
| BG | BN12 | 593 | 4.02 | 0.68 | 0.44 |
| BG | BN13 | 639 | 5.01 | 0.82 | 0.54 |
| BG | BN14 | 380 | 4.14 | 0.71 | 0.48 |
| BG | BN15 | 421 | 4.91 | 0.79 | 0.56 |
| BG | BN16 | 531 | 4.53 | 0.75 | 0.50 |
| BG | BN17 | 761 | 5.26 | 0.85 | 0.55 |
| BG | BS01 | 331 | 4.97 | 0.83 | 0.59 |
| BG | BS02 | 522 | 5.36 | 0.86 | 0.59 |
| BG | BS03 | 419 | 4.08 | 0.69 | 0.47 |
| BG | BS04 | 396 | 4.91 | 0.82 | 0.57 |
| BG | BS05 | 361 | 4.31 | 0.76 | 0.51 |
| **Sea area** | **Station** | **Richness** | **Shannon** | **Simpson** | **Pielou** |
| BG | BS06 | 344 | 4.31 | 0.75 | 0.51 |
| BG | BS07 | 353 | 4.76 | 0.80 | 0.56 |
| BG | BS08 | 315 | 4.70 | 0.80 | 0.57 |
| BG | BS09 | 609 | 5.53 | 0.90 | 0.60 |
| BG | BS10 | 324 | 5.17 | 0.87 | 0.62 |
| BG | BS11 | 176 | 4.31 | 0.80 | 0.58 |
| BG | BS12 | 314 | 5.50 | 0.90 | 0.66 |
| BG | BS13 | 367 | 4.99 | 0.85 | 0.59 |
| BG | BS14 | 424 | 5.31 | 0.88 | 0.61 |
| BG | QZ01 | 1294 | 8.53 | 0.99 | 0.83 |
| BG | QZ02 | 830 | 6.46 | 0.95 | 0.67 |
| BG | QZ03 | 531 | 5.03 | 0.86 | 0.56 |
| BG | QZ04 | 351 | 3.90 | 0.73 | 0.46 |
| BG | QZ05 | 466 | 4.65 | 0.75 | 0.52 |
| BG | QZ06 | 451 | 4.59 | 0.73 | 0.52 |
| **Average** | | **468.56** | **5.06** | **0.83** | **0.58** |
| WS | WS01 | 241 | 4.33 | 0.77 | 0.55 |
| WS | WS02 | 466 | 5.10 | 0.84 | 0.58 |
| WS | WS03 | 284 | 3.93 | 0.72 | 0.48 |
| WS | WS04 | 271 | 3.95 | 0.71 | 0.49 |
| WS | WS05 | 168 | 4.37 | 0.81 | 0.59 |
| WS | WS06 | 320 | 4.02 | 0.74 | 0.48 |
| WS | WS07 | 308 | 4.89 | 0.87 | 0.59 |
| WS | WS08 | 827 | 5.96 | 0.93 | 0.61 |
| WS | WS09 | 96 | 4.02 | 0.79 | 0.61 |
| WS | WS10 | 288 | 4.18 | 0.76 | 0.51 |
| WS | WS11 | 369 | 5.02 | 0.85 | 0.59 |
| WS | WS12 | 425 | 4.66 | 0.80 | 0.53 |
| WS | WS13 | 67 | 3.28 | 0.69 | 0.54 |
| WS | WS14 | 425 | 5.39 | 0.89 | 0.62 |
| WS | WS15 | 332 | 4.42 | 0.79 | 0.53 |
| WS | WS16 | 186 | 4.16 | 0.78 | 0.55 |
| WS | WS17 | 299 | 5.31 | 0.91 | 0.65 |
| WS | WS18 | 413 | 4.45 | 0.77 | 0.51 |
| WS | WS19 | 414 | 4.87 | 0.83 | 0.56 |
| WS | WS20 | 248 | 4.08 | 0.73 | 0.51 |
| WS | WS21 | 439 | 5.72 | 0.91 | 0.65 |
| WS | WS22 | 353 | 5.87 | 0.94 | 0.69 |
| WS | WS23 | 305 | 4.83 | 0.83 | 0.59 |
| WS | WS24 | 427 | 5.09 | 0.85 | 0.58 |
| WS | EH01 | 664 | 6.29 | 0.94 | 0.67 |
| WS | EH02 | 322 | 4.82 | 0.85 | 0.58 |
| WS | EH03 | 428 | 5.29 | 0.86 | 0.61 |
| **Sea area** | **Station** | **Richness** | **Shannon** | **Simpson** | **Pielou** |
| WS | EH04 | 402 | 5.26 | 0.89 | 0.61 |
| WS | EH05 | 485 | 5.75 | 0.93 | 0.64 |
| WS | EH06 | 427 | 5.78 | 0.92 | 0.66 |
| WS | EH07 | 376 | 4.50 | 0.78 | 0.53 |
| WS | EH08 | 443 | 5.58 | 0.92 | 0.63 |
| WS | EH09 | 408 | 5.14 | 0.88 | 0.59 |
| WS | EH10 | 367 | 5.71 | 0.89 | 0.67 |
| WS | EH11 | 470 | 6.02 | 0.93 | 0.68 |
| WS | EH12 | 441 | 6.13 | 0.94 | 0.70 |
| WS | EH13 | 174 | 5.21 | 0.90 | 0.70 |
| WS | EH14 | 514 | 6.02 | 0.92 | 0.67 |
| WS | EH15 | 565 | 4.99 | 0.86 | 0.55 |
| **Average** | | **370.69** | **4.98** | **0.84** | **0.59** |
| **Minimum value of all samples** | | **67** | **3.09** | **0.63** | **0.39** |
| **Maximum value of all samples** | | **1294** | **8.53** | **0.99** | **0.83** |

Table S2 Permutational Multivariate Analysis of Variance (PERMANOVA) to evaluate the distances between groups in the Non-metric Multidimensional Scaling (NMDS) representation of environmental factors.

| **pairs** | **Df** | **SumsOfSqs** | **F.Model** | **R^2^** | ***p*.value** | ***p*.adjusted** |
| --- | --- | --- | --- | --- | --- | --- |
| GD vs BG | 1 | 0.103 | 89.727 | 0.481 | 0.001 | 0.001 |
| GD vs WS | 1 | 0.584 | 82.246 | 0.495 | 0.001 | 0.001 |
| BG vs WS | 1 | 0.298 | 40.691 | 0.314 | 0.001 | 0.001 |

Table S3 Permutational Multivariate Analysis of Variance (PERMANOVA) to evaluate the distances between groups in the Non-metric Multidimensional Scaling (NMDS) representation of bacterial communities.

| **pairs** | **Df** | **SumsOfSqs** | **F.Model** | **R^2^** | ***p*.value** | ***p*.adjusted** |
| --- | --- | --- | --- | --- | --- | --- |
| GD vs BG | 1 | 4.236 | 18.212 | 0.158 | 0.001 | 0.001 |
| GD vs WS | 1 | 5.162 | 24.144 | 0.223 | 0.001 | 0.001 |
| BG vs WS | 1 | 3.073 | 15.395 | 0.147 | 0.001 | 0.001 |

Table S4 The assembly mechanism of each bacterial bin.

| **Bin** | **BinRA** | **HoS** | **HeS** | **HD** | **DL** | **DR** | **TopTaxonID** | **Phylum** | **Class** |
| --- | --- | --- | --- | --- | --- | --- | --- | --- | --- |
| bin1 | 45.70 | 100.00 | 0.00 | 0.00 | 0.00 | 0.00 | ASV1 | Cyanobacteriota | Cyanophyceae |
| bin2 | 0.03 | 6.69 | 2.54 | 0.02 | 86.19 | 4.55 | ASV11406 | Cyanobacteriota | Cyanophyceae |
| bin3 | 0.02 | 8.93 | 2.20 | 0.00 | 82.24 | 6.62 | ASV9343 | Cyanobacteriota | Cyanophyceae |
| bin4 | 0.02 | 4.29 | 1.56 | 0.00 | 87.13 | 7.01 | ASV4400 | Cyanobacteriota | Cyanophyceae |
| bin5 | 0.03 | 1.48 | 2.10 | 0.00 | 90.63 | 5.79 | ASV8575 | Bacteroidota | Flavobacteriia |
| bin6 | 0.03 | 5.59 | 0.98 | 0.00 | 88.03 | 5.40 | ASV19152 | Pseudomonadota | Alphaproteobacteria |
| bin7 | 0.06 | 9.67 | 26.71 | 0.00 | 44.81 | 18.81 | ASV3422 | Pseudomonadota | Epsilonproteobacteria |
| bin8 | 0.44 | 80.40 | 0.75 | 0.18 | 0.30 | 18.37 | ASV521 | SAR406_clade | unclassified |
| bin9 | 0.10 | 26.77 | 1.17 | 0.00 | 46.53 | 25.53 | ASV532 | SAR406_clade | unclassified |
| bin10 | 0.27 | 56.57 | 0.64 | 0.08 | 14.85 | 27.86 | ASV480 | Verrucomicrobiota | Verrucomicrobiia |
| bin11 | 0.46 | 7.96 | 0.09 | 0.20 | 72.86 | 18.88 | ASV1615 | Bacteroidota | Saprospiria |
| bin12 | 0.09 | 62.87 | 0.83 | 0.00 | 10.25 | 26.05 | ASV1174 | Bacteroidota | Chitinophagia |
| bin13 | 0.03 | 15.61 | 0.00 | 0.00 | 71.77 | 12.62 | ASV5628 | Bacteroidota | Saprospiria |
| bin14 | 0.14 | 22.98 | 3.84 | 0.00 | 50.29 | 22.89 | ASV84 | Bacteroidota | Saprospiria |
| bin15 | 0.07 | 17.31 | 0.45 | 0.05 | 57.79 | 24.41 | ASV2041 | Bacteroidota | Cytophagia |
| bin16 | 0.05 | 9.92 | 8.80 | 0.00 | 76.74 | 4.53 | ASV2603 | Bacteroidota | Bacteroidia |
| bin17 | 0.25 | 22.40 | 1.47 | 0.02 | 53.09 | 23.03 | ASV124 | Bacteroidota | Sphingobacteriia |
| bin18 | 0.95 | 88.57 | 0.87 | 0.43 | 0.00 | 10.14 | ASV45 | Bacteroidota | Flavobacteriia |
| bin19 | 2.56 | 42.31 | 0.27 | 2.28 | 8.32 | 46.81 | ASV21 | Bacteroidota | Flavobacteriia |
| bin20 | 0.30 | 3.32 | 0.07 | 0.16 | 79.44 | 17.01 | ASV258 | Bacteroidota | Flavobacteriia |
| bin21 | 0.14 | 9.35 | 1.76 | 0.05 | 72.13 | 16.70 | ASV1834 | Bacteroidota | Flavobacteriia |
| bin22 | 0.09 | 18.82 | 0.86 | 0.00 | 65.80 | 14.51 | ASV1080 | Bacteroidota | Flavobacteriia |
| bin23 | 0.29 | 28.68 | 4.34 | 0.13 | 50.06 | 16.79 | ASV210 | Bacteroidota | Flavobacteriia |
| bin24 | 0.19 | 32.38 | 4.74 | 0.07 | 40.85 | 21.96 | ASV1140 | Bacteroidota | Flavobacteriia |
| bin25 | 0.26 | 21.08 | 0.83 | 0.37 | 18.54 | 59.18 | ASV1127 | Bacteroidota | Flavobacteriia |
| bin26 | 0.30 | 19.93 | 0.88 | 0.11 | 23.39 | 55.69 | ASV1132 | Bacteroidota | Flavobacteriia |
| bin27 | 0.08 | 35.24 | 1.48 | 0.02 | 18.39 | 44.88 | ASV1476 | Dadaibacteriota | Dadabacteriia |
| bin28 | 0.41 | 92.80 | 3.13 | 0.17 | 1.07 | 2.84 | ASV270 | Bacteroidota | Flavobacteriia |
| **Bin** | **BinRA** | **HoS** | **HeS** | **HD** | **DL** | **DR** | **TopTaxonID** | **Phylum** | **Class** |
| bin29 | 0.02 | 20.85 | 17.72 | 0.00 | 50.35 | 11.07 | ASV12771 | Bacteroidota | Flavobacteriia |
| bin30 | 0.03 | 1.70 | 0.00 | 0.00 | 91.08 | 7.22 | ASV19146 | Pseudomonadota | Alphaproteobacteria |
| bin31 | 0.02 | 1.00 | 1.36 | 0.00 | 88.02 | 9.62 | ASV17513 | Pseudomonadota | Gammaproteobacteria |
| bin32 | 2.03 | 10.41 | 8.15 | 4.59 | 23.20 | 53.66 | ASV18 | Bacteroidota | Flavobacteriia |
| bin33 | 0.55 | 62.25 | 2.19 | 0.02 | 21.71 | 13.83 | ASV827 | Bacteroidota | Flavobacteriia |
| bin34 | 0.67 | 85.13 | 2.78 | 0.83 | 2.24 | 9.02 | ASV672 | Bacteroidota | Flavobacteriia |
| bin35 | 0.54 | 54.89 | 0.96 | 0.08 | 34.49 | 9.58 | ASV19 | Bacteroidota | Rhodothermia |
| bin36 | 0.07 | 2.02 | 0.31 | 0.00 | 81.15 | 16.53 | ASV17690 | Cyanobacteriota | Cyanophyceae |
| bin37 | 0.38 | 5.27 | 2.11 | 0.01 | 8.71 | 83.90 | ASV11 | Planctomycetota | Phycisphaerae |
| bin38 | 0.13 | 67.50 | 4.69 | 0.10 | 11.90 | 15.82 | ASV128 | Planctomycetota | Phycisphaerae |
| bin39 | 0.29 | 28.72 | 5.52 | 0.31 | 48.33 | 17.13 | ASV518 | Pseudomonadota | Gammaproteobacteria |
| bin40 | 0.08 | 18.32 | 0.64 | 0.03 | 43.82 | 37.19 | ASV2052 | Pseudomonadota | Gammaproteobacteria |
| bin41 | 0.01 | 1.44 | 9.81 | 0.00 | 76.48 | 12.27 | ASV23374 | Pseudomonadota | Gammaproteobacteria |
| bin42 | 0.16 | 77.93 | 8.22 | 0.05 | 6.05 | 7.75 | ASV48 | Pseudomonadota | Gammaproteobacteria |
| bin43 | 0.17 | 62.01 | 1.80 | 0.08 | 9.19 | 26.92 | ASV81 | Pseudomonadota | Betaproteobacteria |
| bin44 | 0.07 | 8.19 | 3.43 | 0.00 | 79.64 | 8.73 | ASV12764 | Pseudomonadota | Betaproteobacteria |
| bin45 | 0.07 | 32.47 | 2.53 | 0.00 | 45.34 | 19.65 | ASV702 | Pseudomonadota | Betaproteobacteria |
| bin46 | 0.36 | 2.97 | 0.18 | 0.60 | 73.26 | 22.99 | ASV73 | Pseudomonadota | Betaproteobacteria |
| bin47 | 0.00 | 0.48 | 0.89 | 0.00 | 79.21 | 19.42 | ASV19466 | Pseudomonadota | Alphaproteobacteria |
| bin48 | 0.01 | 5.54 | 0.18 | 0.00 | 81.85 | 12.42 | ASV17517 | Bacteroidota | Flavobacteriia |
| bin49 | 0.02 | 1.63 | 1.48 | 0.00 | 91.07 | 5.82 | ASV9668 | Pseudomonadota | Alphaproteobacteria |
| bin50 | 0.01 | 0.88 | 0.21 | 0.00 | 90.84 | 8.07 | ASV23193 | Pseudomonadota | Gammaproteobacteria |
| bin51 | 0.15 | 60.67 | 7.65 | 0.00 | 11.78 | 19.90 | ASV828 | Pseudomonadota | Gammaproteobacteria |
| bin52 | 0.06 | 37.11 | 11.74 | 0.00 | 37.04 | 14.12 | ASV3499 | Pseudomonadota | Gammaproteobacteria |
| bin53 | 0.09 | 55.39 | 1.92 | 0.04 | 5.86 | 36.79 | ASV1148 | Pseudomonadota | Gammaproteobacteria |
| bin54 | 0.01 | 10.48 | 4.83 | 0.00 | 71.14 | 13.56 | ASV1786 | Pseudomonadota | Gammaproteobacteria |
| bin55 | 0.02 | 10.04 | 3.79 | 0.01 | 68.00 | 18.15 | ASV132 | Pseudomonadota | Gammaproteobacteria |
| bin56 | 0.03 | 10.98 | 10.16 | 0.00 | 55.64 | 23.21 | ASV2010 | Pseudomonadota | Gammaproteobacteria |
| bin57 | 0.02 | 8.89 | 0.28 | 0.00 | 74.40 | 16.42 | ASV6887 | Pseudomonadota | Gammaproteobacteria |
| **Bin** | **BinRA** | **HoS** | **HeS** | **HD** | **DL** | **DR** | **TopTaxonID** | **Phylum** | **Class** |
| bin58 | 0.03 | 13.59 | 2.11 | 0.00 | 55.52 | 28.78 | ASV1159 | Pseudomonadota | Gammaproteobacteria |
| bin59 | 0.02 | 8.77 | 3.11 | 0.00 | 63.36 | 24.76 | ASV1378 | Pseudomonadota | Gammaproteobacteria |
| bin60 | 0.07 | 9.31 | 1.20 | 0.08 | 48.46 | 40.95 | ASV1643 | Pseudomonadota | Gammaproteobacteria |
| bin61 | 0.14 | 61.16 | 2.58 | 0.06 | 12.27 | 23.93 | ASV130 | Pseudomonadota | Gammaproteobacteria |
| bin62 | 0.07 | 26.94 | 5.33 | 0.00 | 54.54 | 13.19 | ASV1170 | Pseudomonadota | Gammaproteobacteria |
| bin63 | 0.21 | 20.26 | 4.02 | 0.17 | 60.01 | 15.53 | ASV1220 | Pseudomonadota | Gammaproteobacteria |
| bin64 | 0.06 | 3.06 | 0.94 | 0.00 | 78.63 | 17.37 | ASV633 | Pseudomonadota | Gammaproteobacteria |
| bin65 | 0.01 | 0.22 | 0.14 | 0.00 | 88.55 | 11.09 | ASV15616 | SAR324_clade | unclassified |
| bin66 | 0.39 | 30.08 | 1.35 | 0.57 | 30.69 | 37.31 | ASV1754 | Pseudomonadota | Gammaproteobacteria |
| bin67 | 0.01 | 0.81 | 0.28 | 0.00 | 92.26 | 6.65 | ASV21336 | Pseudomonadota | Gammaproteobacteria |
| bin68 | 0.43 | 33.80 | 0.57 | 0.39 | 7.78 | 57.46 | ASV23 | Pseudomonadota | Gammaproteobacteria |
| bin69 | 1.84 | 97.01 | 0.51 | 0.30 | 0.58 | 1.61 | ASV6 | Pseudomonadota | Gammaproteobacteria |
| bin70 | 0.01 | 1.26 | 1.66 | 0.00 | 91.71 | 5.37 | ASV21328 | Pseudomonadota | Betaproteobacteria |
| bin71 | 0.14 | 58.89 | 1.40 | 0.00 | 8.44 | 31.27 | ASV61 | Pseudomonadota | Gammaproteobacteria |
| bin72 | 0.50 | 86.60 | 4.59 | 0.06 | 0.00 | 8.75 | ASV85 | Pseudomonadota | Gammaproteobacteria |
| bin73 | 0.01 | 4.44 | 0.30 | 0.00 | 86.20 | 9.06 | ASV3251 | Pseudomonadota | Gammaproteobacteria |
| bin74 | 0.66 | 88.29 | 4.79 | 0.16 | 0.03 | 6.72 | ASV647 | Pseudomonadota | Gammaproteobacteria |
| bin75 | 0.02 | 2.49 | 1.45 | 0.00 | 88.57 | 7.49 | ASV21325 | Pseudomonadota | Gammaproteobacteria |
| bin76 | 0.01 | 2.12 | 0.87 | 0.00 | 87.37 | 9.65 | ASV11811 | Pseudomonadota | Gammaproteobacteria |
| bin77 | 0.22 | 69.94 | 0.47 | 1.01 | 1.28 | 27.30 | ASV147 | Pseudomonadota | Gammaproteobacteria |
| bin78 | 0.37 | 7.83 | 0.39 | 0.40 | 7.49 | 83.89 | ASV10 | Pseudomonadota | Gammaproteobacteria |
| bin79 | 0.45 | 33.09 | 0.02 | 0.19 | 3.71 | 62.98 | ASV33 | Pseudomonadota | Gammaproteobacteria |
| bin80 | 0.03 | 17.74 | 0.20 | 0.00 | 63.15 | 18.90 | ASV2101 | Pseudomonadota | Gammaproteobacteria |
| bin81 | 0.01 | 3.35 | 1.16 | 0.00 | 84.29 | 11.20 | ASV4284 | Pseudomonadota | Gammaproteobacteria |
| bin82 | 0.19 | 48.09 | 3.25 | 0.11 | 23.38 | 25.17 | ASV1355 | Pseudomonadota | Gammaproteobacteria |
| bin83 | 0.19 | 32.92 | 2.07 | 0.43 | 38.90 | 25.68 | ASV89 | Pseudomonadota | Gammaproteobacteria |
| bin84 | 0.70 | 82.67 | 5.08 | 0.21 | 5.60 | 6.44 | ASV486 | Pseudomonadota | Gammaproteobacteria |
| bin85 | 0.04 | 5.77 | 0.60 | 0.00 | 42.97 | 50.66 | ASV1193 | Pseudomonadota | Gammaproteobacteria |
| bin86 | 1.16 | 91.10 | 0.07 | 0.49 | 0.24 | 8.09 | ASV24 | Pseudomonadota | Gammaproteobacteria |
| **Bin** | **BinRA** | **HoS** | **HeS** | **HD** | **DL** | **DR** | **TopTaxonID** | **Phylum** | **Class** |
| bin87 | 0.35 | 67.77 | 0.78 | 0.09 | 14.74 | 16.62 | ASV2291 | Pseudomonadota | Gammaproteobacteria |
| bin88 | 0.02 | 27.66 | 2.83 | 0.00 | 47.83 | 21.68 | ASV5659 | Pseudomonadota | Gammaproteobacteria |
| bin89 | 0.02 | 7.67 | 2.61 | 0.00 | 63.09 | 26.62 | ASV3553 | Pseudomonadota | Gammaproteobacteria |
| bin90 | 0.16 | 68.10 | 1.39 | 0.17 | 4.52 | 25.82 | ASV1356 | Pseudomonadota | Gammaproteobacteria |
| bin91 | 1.80 | 90.98 | 0.54 | 0.34 | 1.23 | 6.91 | ASV55 | Pseudomonadota | Gammaproteobacteria |
| bin92 | 0.01 | 5.64 | 0.41 | 0.00 | 76.21 | 17.74 | ASV1455 | Pseudomonadota | Gammaproteobacteria |
| bin93 | 0.02 | 4.16 | 2.59 | 0.00 | 88.65 | 4.60 | ASV17147 | Pseudomonadota | Alphaproteobacteria |
| bin94 | 0.11 | 0.82 | 0.06 | 0.00 | 94.85 | 4.27 | ASV15605 | Cyanobacteriota | Cyanophyceae |
| bin95 | 0.00 | 7.05 | 0.04 | 0.00 | 75.29 | 17.63 | ASV18890 | Bacillota | Bacilli |
| bin96 | 0.01 | 0.57 | 0.58 | 0.00 | 92.76 | 6.09 | ASV15260 | Pseudomonadota | Alphaproteobacteria |
| bin97 | 0.02 | 7.70 | 1.34 | 0.00 | 83.93 | 7.03 | ASV11809 | Cyanobacteriota | Cyanophyceae |
| bin98 | 0.02 | 3.53 | 1.96 | 0.00 | 82.10 | 12.41 | ASV7082 | unclassified | unclassified |
| bin99 | 0.04 | 2.43 | 2.16 | 0.00 | 90.57 | 4.83 | ASV19191 | Cyanobacteriota | Cyanophyceae |
| bin100 | 0.08 | 47.50 | 18.81 | 0.00 | 22.40 | 11.29 | ASV1648 | Bacillota | Negativicutes |
| bin101 | 0.01 | 1.94 | 1.12 | 0.00 | 88.24 | 8.69 | ASV21327 | Pseudomonadota | Alphaproteobacteria |
| bin102 | 0.02 | 0.41 | 0.09 | 0.00 | 95.74 | 3.76 | ASV15609 | Pseudomonadota | Alphaproteobacteria |
| bin103 | 0.01 | 1.96 | 1.46 | 0.00 | 81.08 | 15.50 | ASV22081 | Pseudomonadota | Gammaproteobacteria |
| bin104 | 0.01 | 1.06 | 1.01 | 0.00 | 89.75 | 8.19 | ASV24060 | Pseudomonadota | Gammaproteobacteria |
| bin105 | 0.02 | 5.49 | 5.42 | 0.00 | 81.78 | 7.30 | ASV20503 | Bacteroidota | Flavobacteriia |
| bin106 | 0.50 | 70.14 | 0.49 | 0.36 | 6.69 | 22.32 | ASV227 | Bacteroidota | Flavobacteriia |
| bin107 | 0.47 | 24.01 | 0.88 | 0.26 | 32.71 | 42.14 | ASV97 | Pseudomonadota | Gammaproteobacteria |
| bin108 | 0.09 | 5.22 | 3.10 | 0.00 | 65.15 | 26.52 | ASV516 | Pseudomonadota | Gammaproteobacteria |
| bin109 | 0.14 | 33.93 | 0.69 | 0.34 | 50.77 | 14.27 | ASV1146 | Pseudomonadota | Gammaproteobacteria |
| bin110 | 0.03 | 6.00 | 1.07 | 0.00 | 86.57 | 6.36 | ASV17142 | Pseudomonadota | Alphaproteobacteria |
| bin111 | 1.07 | 30.51 | 0.06 | 2.84 | 0.03 | 66.56 | ASV38 | Pseudomonadota | Alphaproteobacteria |
| bin112 | 0.01 | 1.13 | 3.20 | 0.00 | 83.42 | 12.24 | ASV16646 | Pseudomonadota | Alphaproteobacteria |
| bin113 | 0.08 | 29.35 | 0.58 | 0.00 | 0.77 | 69.31 | ASV612 | Pseudomonadota | Alphaproteobacteria |
| bin114 | 0.01 | 2.85 | 6.57 | 0.00 | 81.68 | 8.90 | ASV1959 | Bacteroidota | Flavobacteriia |
| bin115 | 0.04 | 1.18 | 0.54 | 0.00 | 70.84 | 27.44 | ASV1632 | Pseudomonadota | Alphaproteobacteria |
| **Bin** | **BinRA** | **HoS** | **HeS** | **HD** | **DL** | **DR** | **TopTaxonID** | **Phylum** | **Class** |
| bin116 | 0.58 | 85.61 | 0.90 | 0.28 | 1.80 | 11.40 | ASV79 | Pseudomonadota | Alphaproteobacteria |
| bin117 | 1.13 | 97.27 | 0.91 | 0.04 | 0.00 | 1.77 | ASV15 | Pseudomonadota | Alphaproteobacteria |
| bin118 | 0.03 | 4.25 | 7.71 | 0.00 | 66.22 | 21.81 | ASV1272 | unclassified | unclassified |
| bin119 | 0.40 | 78.22 | 2.97 | 0.23 | 8.54 | 10.04 | ASV176 | Pseudomonadota | Alphaproteobacteria |
| bin120 | 3.10 | 97.82 | 0.00 | 0.09 | 0.00 | 2.10 | ASV3 | Pseudomonadota | Alphaproteobacteria |
| bin121 | 0.00 | 1.74 | 0.52 | 0.00 | 84.86 | 12.88 | ASV17154 | Pseudomonadota | Alphaproteobacteria |
| bin122 | 0.02 | 1.46 | 0.33 | 0.00 | 90.18 | 8.03 | ASV19199 | Pseudomonadota | Alphaproteobacteria |
| bin123 | 0.62 | 22.85 | 1.12 | 0.36 | 15.90 | 59.77 | ASV1126 | Pseudomonadota | Alphaproteobacteria |
| bin124 | 0.35 | 65.64 | 1.62 | 0.14 | 0.20 | 32.39 | ASV53 | Pseudomonadota | Alphaproteobacteria |
| bin125 | 0.91 | 89.51 | 0.72 | 0.64 | 0.04 | 9.09 | ASV41 | Pseudomonadota | Alphaproteobacteria |
| bin126 | 0.02 | 6.25 | 2.79 | 0.00 | 59.89 | 31.08 | ASV256 | Pseudomonadota | Alphaproteobacteria |
| bin127 | 0.40 | 39.72 | 1.17 | 0.00 | 0.22 | 58.88 | ASV58 | Pseudomonadota | Alphaproteobacteria |
| bin128 | 0.08 | 29.14 | 1.15 | 0.00 | 14.24 | 55.47 | ASV178 | Pseudomonadota | Alphaproteobacteria |
| bin129 | 0.21 | 65.65 | 9.97 | 0.06 | 10.03 | 14.28 | ASV865 | Bacillota | Bacilli |
| bin130 | 0.02 | 8.19 | 0.46 | 0.00 | 83.77 | 7.58 | ASV23367 | Pseudomonadota | Alphaproteobacteria |
| bin131 | 0.01 | 2.17 | 0.23 | 0.00 | 86.37 | 11.23 | ASV5171 | unclassified | unclassified |
| bin132 | 0.12 | 49.56 | 4.92 | 0.01 | 3.25 | 42.25 | ASV117 | Pseudomonadota | Alphaproteobacteria |
| bin133 | 0.38 | 1.34 | 0.67 | 0.64 | 82.30 | 15.05 | ASV1621 | Pseudomonadota | Alphaproteobacteria |
| bin134 | 0.57 | 60.04 | 0.13 | 0.10 | 9.59 | 30.14 | ASV135 | Pseudomonadota | Alphaproteobacteria |
| bin135 | 0.01 | 7.42 | 0.66 | 0.00 | 84.21 | 7.71 | ASV19156 | Pseudomonadota | Alphaproteobacteria |
| bin136 | 4.52 | 37.13 | 0.00 | 15.41 | 8.24 | 39.22 | ASV154 | Pseudomonadota | Alphaproteobacteria |
| bin137 | 0.17 | 15.64 | 0.99 | 0.05 | 52.48 | 30.84 | ASV494 | Pseudomonadota | Alphaproteobacteria |
| bin138 | 0.03 | 15.29 | 12.33 | 0.00 | 58.09 | 14.28 | ASV1755 | Pseudomonadota | Alphaproteobacteria |
| bin139 | 0.07 | 13.49 | 16.80 | 0.01 | 61.65 | 8.05 | ASV3723 | Pseudomonadota | Alphaproteobacteria |
| bin140 | 0.27 | 62.12 | 1.39 | 0.14 | 16.28 | 20.08 | ASV201 | Pseudomonadota | Alphaproteobacteria |
| bin141 | 0.12 | 29.40 | 0.30 | 0.04 | 30.45 | 39.81 | ASV561 | Pseudomonadota | Alphaproteobacteria |
| bin142 | 0.61 | 7.50 | 0.44 | 0.75 | 61.61 | 29.69 | ASV615 | Pseudomonadota | Alphaproteobacteria |
| bin143 | 0.24 | 52.92 | 0.17 | 0.32 | 10.24 | 36.35 | ASV1347 | Pseudomonadota | Alphaproteobacteria |
| bin144 | 0.06 | 8.41 | 0.06 | 0.00 | 48.59 | 42.94 | ASV1403 | Pseudomonadota | Alphaproteobacteria |
| **Bin** | **BinRA** | **HoS** | **HeS** | **HD** | **DL** | **DR** | **TopTaxonID** | **Phylum** | **Class** |
| bin145 | 0.18 | 46.20 | 0.29 | 0.00 | 1.35 | 52.16 | ASV509 | Pseudomonadota | Alphaproteobacteria |
| bin146 | 0.94 | 95.77 | 0.22 | 0.90 | 0.00 | 3.10 | ASV28 | Pseudomonadota | Alphaproteobacteria |
| bin147 | 0.02 | 2.80 | 2.55 | 0.00 | 87.74 | 6.91 | ASV18542 | Pseudomonadota | Alphaproteobacteria |
| bin148 | 0.18 | 27.71 | 8.40 | 0.18 | 48.24 | 15.48 | ASV1644 | Pseudomonadota | Alphaproteobacteria |
| bin149 | 0.05 | 12.94 | 7.32 | 0.08 | 66.28 | 13.38 | ASV495 | Pseudomonadota | Oligoflexia |
| bin150 | 0.01 | 5.55 | 0.64 | 0.00 | 73.53 | 20.27 | ASV1250 | Pseudomonadota | Oligoflexia |
| bin151 | 0.09 | 0.72 | 1.39 | 0.15 | 80.23 | 17.51 | ASV2633 | Pseudomonadota | Bdellovibrionia |
| bin152 | 0.11 | 12.89 | 0.40 | 0.56 | 35.23 | 50.92 | ASV1438 | Pseudomonadota | Bdellovibrionia |
| bin153 | 0.07 | 4.93 | 0.00 | 0.04 | 32.47 | 62.56 | ASV1178 | Pseudomonadota | Bdellovibrionia |
| bin154 | 0.04 | 3.65 | 1.64 | 0.04 | 71.17 | 23.51 | ASV387 | unclassified | unclassified |
| bin155 | 0.08 | 12.06 | 0.00 | 0.05 | 56.65 | 31.23 | ASV1192 | Pseudomonadota | Desulfuromonadia |
| bin156 | 0.02 | 11.13 | 33.35 | 0.00 | 51.84 | 3.68 | ASV7254 | unclassified | unclassified |
| bin157 | 0.06 | 20.71 | 9.71 | 0.11 | 56.95 | 12.52 | ASV1718 | Pseudomonadota | Desulfuromonadia |
| bin158 | 0.02 | 14.36 | 15.06 | 0.00 | 63.04 | 7.54 | ASV7034 | Pseudomonadota | Polyangia |
| bin159 | 0.04 | 13.42 | 3.06 | 0.00 | 61.43 | 22.09 | ASV328 | Pseudomonadota | Myxococcia |
| bin160 | 0.01 | 8.06 | 0.26 | 0.00 | 80.82 | 10.86 | ASV5658 | Pseudomonadota | Polyangia |
| bin161 | 0.03 | 7.93 | 0.90 | 0.00 | 83.40 | 7.76 | ASV7064 | Pseudomonadota | Alphaproteobacteria |
| bin162 | 0.41 | 0.21 | 0.00 | 0.41 | 9.83 | 89.55 | ASV56 | SAR324_clade | unclassified |
| bin163 | 0.02 | 12.24 | 4.95 | 0.00 | 74.63 | 8.18 | ASV818 | Bacteroidota | Kapabacteria |
| bin164 | 0.04 | 25.39 | 11.10 | 0.00 | 54.49 | 9.02 | ASV2543 | Bacillota | Negativicutes |
| bin165 | 0.18 | 30.00 | 1.89 | 0.06 | 44.95 | 23.10 | ASV39 | Bacillota | Bacilli |
| bin166 | 0.35 | 67.01 | 1.36 | 0.16 | 12.46 | 19.02 | ASV75 | Bacillota | Bacilli |
| bin167 | 0.02 | 7.01 | 0.00 | 0.00 | 79.68 | 13.31 | ASV6976 | Bacillota | Clostridia |
| bin168 | 0.05 | 4.99 | 5.89 | 0.00 | 81.98 | 7.14 | ASV4479 | Bacillota | Clostridia |
| bin169 | 0.08 | 13.19 | 25.71 | 0.00 | 53.67 | 7.43 | ASV4176 | Fusobacteriota | Fusobacteriia |
| bin170 | 0.04 | 22.65 | 3.69 | 0.00 | 57.94 | 15.72 | ASV2173 | Altimarinota | Gracilibacteria |
| bin171 | 0.48 | 68.64 | 1.10 | 0.75 | 5.90 | 23.62 | ASV207 | unclassified | unclassified |
| bin172 | 0.17 | 58.31 | 2.38 | 0.01 | 10.67 | 28.64 | ASV504 | Chloroflexota | Dehalococcoidia |
| bin173 | 0.12 | 47.68 | 0.59 | 0.07 | 37.01 | 14.65 | ASV525 | SAR406_clade | unclassified |
| **Bin** | **BinRA** | **HoS** | **HeS** | **HD** | **DL** | **DR** | **TopTaxonID** | **Phylum** | **Class** |
| bin174 | 0.02 | 3.23 | 33.99 | 0.00 | 58.50 | 4.28 | ASV7018 | Chloroflexota | KD4-96 |
| bin175 | 0.01 | 6.52 | 12.63 | 0.00 | 76.57 | 4.28 | ASV838 | Thermomicrobiota | Thermomicrobia |
| bin176 | 0.03 | 70.29 | 0.97 | 0.00 | 25.09 | 3.66 | ASV7035 | Chloroflexota | Anaerolineae |
| bin177 | 0.12 | 28.75 | 5.40 | 0.00 | 49.56 | 16.29 | ASV547 | Chloroflexota | Dehalococcoidia |
| bin178 | 0.03 | 8.13 | 28.47 | 0.00 | 58.17 | 5.23 | ASV7001 | Acidobacteriota | Vicinamibacteria |
| bin179 | 0.03 | 7.66 | 3.28 | 0.00 | 86.04 | 3.02 | ASV3529 | Acidobacteriota | Thermoanaerobaculia |
| bin180 | 0.03 | 52.92 | 23.28 | 0.00 | 14.15 | 9.64 | ASV8669 | Pseudomonadota | Desulfuromonadia |
| bin181 | 0.04 | 11.02 | 0.44 | 0.01 | 76.34 | 12.20 | ASV1203 | Pseudomonadota | Desulfuromonadia |
| bin182 | 0.02 | 4.19 | 0.29 | 0.00 | 78.10 | 17.42 | ASV8666 | PAUC34f | unclassified |
| bin183 | 0.12 | 65.81 | 1.20 | 0.11 | 12.49 | 20.39 | ASV274 | Planctomycetota | OM190 |
| bin184 | 0.17 | 44.38 | 1.84 | 0.12 | 8.45 | 45.20 | ASV67 | Verrucomicrobiota | Verrucomicrobiae |
| bin185 | 0.09 | 22.30 | 0.05 | 0.01 | 48.70 | 28.94 | ASV112 | Verrucomicrobiota | Verrucomicrobiae |
| bin186 | 0.04 | 36.04 | 2.48 | 0.00 | 40.77 | 20.71 | ASV313 | Verrucomicrobiota | Kiritimatiellae |
| bin187 | 0.07 | 11.81 | 1.15 | 0.11 | 72.02 | 14.91 | ASV452 | Planctomycetota | OM190 |
| bin188 | 0.02 | 10.90 | 27.03 | 0.00 | 52.32 | 9.74 | ASV7813 | Planctomycetota | Planctomycetia |
| bin189 | 0.46 | 93.68 | 0.02 | 0.25 | 0.03 | 6.01 | ASV110 | Actinomycetota | Acidimicrobiia |
| bin190 | 0.22 | 11.37 | 0.00 | 0.03 | 52.91 | 35.69 | ASV314 | Actinomycetota | Acidimicrobiia |
| bin191 | 0.56 | 88.51 | 0.85 | 0.30 | 1.33 | 9.02 | ASV507 | Actinomycetota | Acidimicrobiia |
| bin192 | 0.03 | 6.12 | 4.24 | 0.00 | 88.01 | 1.64 | ASV6982 | Actinomycetota | Acidimicrobiia |
| bin193 | 4.96 | 98.79 | 1.13 | 0.01 | 0.00 | 0.07 | ASV7 | Actinomycetota | Acidimicrobiia |
| bin194 | 0.04 | 28.49 | 2.49 | 0.00 | 49.14 | 19.88 | ASV960 | Actinomycetota | Actinobacteria |
| bin195 | 0.44 | 65.12 | 3.47 | 0.10 | 19.14 | 12.17 | ASV791 | Actinomycetota | Actinobacteria |
| bin196 | 0.13 | 35.33 | 1.39 | 0.07 | 48.08 | 15.13 | ASV200 | Actinomycetota | Actinobacteria |
| bin197 | 0.21 | 30.56 | 1.58 | 0.36 | 31.30 | 36.20 | ASV157 | Actinomycetota | Actinobacteria |
| bin198 | 0.04 | 9.46 | 8.46 | 0.01 | 48.68 | 33.39 | ASV242 | Actinomycetota | Actinobacteria |
| bin199 | 0.04 | 14.19 | 3.10 | 0.00 | 70.61 | 12.10 | ASV3310 | Actinomycetota | Thermoleophilia |
| bin200 | 0.14 | 7.17 | 2.40 | 0.00 | 82.96 | 7.47 | ASV1961 | Actinomycetota | Acidimicrobiia |
| bin201 | 0.02 | 10.11 | 34.44 | 0.00 | 30.57 | 24.88 | ASV1984 | Actinomycetota | Acidimicrobiia |
| bin202 | 0.16 | 21.13 | 8.75 | 0.00 | 50.05 | 20.07 | ASV595 | Cyanobacteriota | Cyanobacteriia |
| **Bin** | **BinRA** | **HoS** | **HeS** | **HD** | **DL** | **DR** | **TopTaxonID** | **Phylum** | **Class** |
| bin203 | 0.12 | 3.25 | 2.47 | 0.29 | 77.53 | 16.47 | ASV327 | WPS-2 | unclassified |
| bin204 | 0.07 | 15.73 | 0.52 | 0.33 | 73.65 | 9.77 | ASV8717 | Margulisiibacteriota | unclassified |

Note: BinRA, Relative abundance of Bin; TopTaxonID, Representative ASV ID

Table S5 Comparison of topological characteristics of bacterial co-occurrence networks.

| **Area** | **Nodes** | **Edges** | **AD** | **ND** | **GD** | **MD** | **CC** | **ACC** | **APL** | **NM** | **NPC** | **NNC** |
| --- | --- | --- | --- | --- | --- | --- | --- | --- | --- | --- | --- | --- |
| Total | 1132 | 4535 | 11.414 | 17 | 0.007 | 0.862 | 124 | 0.515 | 5.318 | 138 | 4357 | 178 |
| GD | 748 | 4076 | 15.882 | 24 | 0.015 | 0.687 | 58 | 0.523 | 7.195 | 66 | 3949 | 127 |
| BG | 929 | 5145 | 14.622 | 20 | 0.012 | 0.909 | 59 | 0.543 | 6.097 | 74 | 4716 | 429 |
| WS | 518 | 3168 | 19.104 | 16 | 0.024 | 0.589 | 40 | 0.533 | 4.735 | 47 | 3119 | 49 |

Note: AD, Average degree: ND, Network Diameter; GD, Graph Density; MD, Modularity; CC, Connected Components; ACC, Average Clustering Coefficient; APL, Average Path length; NM, Number of modules; NPC, Number of positive correlations; NNC, Number of negative correlations.

Table S6 Sample information of surface water in the South China Sea

| **Station** | **Area** | **Longitude**  **（°E）** | **Latitude**  **（°N）** | **Date** | **Depth**  **(m)** | **Temperature**  **（℃）** | **Salinity** | **DO**  **(mg/L)** | **NH_4_^+^**  **(μmol/L)** | **NO_3_^-^**  **(μmol/L)** | **NO_2_^-^**  **(μmol/L)** | **SiO_3_^2-^**  **(μmol/L)** | **PO_4_^3-^**  **(μmol/L)** | **Chl *a***  **(μg/L)** |
| --- | --- | --- | --- | --- | --- | --- | --- | --- | --- | --- | --- | --- | --- | --- |
| EG01 | GD | 117.01 | 23.09 | 20200625 | 84 | 30.10 | 31.79 | 7.45 | 1.07 | 0.26 | 0.17 | 2.68 | 0.11 | 0.96 |
| EG02 |  | 117.12 | 22.85 | 20200625 | 28 | 29.54 | 36.13 | 7.21 | 0.89 | 0.24 | 0.18 | 2.62 | 0.09 | 0.62 |
| EG03 |  | 117.22 | 22.6 | 20200625 | 37 | 26.30 | 31.30 | 7.37 | 1.13 | 0.10 | 0.18 | 4.73 | 0.07 | 0.27 |
| EG04 |  | 117.32 | 22.36 | 20200625 | 41 | 26.99 | 34.41 | 7.39 | 1.25 | 0.21 | 0.17 | 0.91 | 0.14 | 0.08 |
| EG05 |  | 117.42 | 22.11 | 20200624 | 40 | 24.36 | 31.36 | 6.69 | 0.93 | 3.57 | 0.38 | 2.38 | 0.14 | 0.37 |
| EG06 |  | 116.35 | 22.83 | 20200624 | 21 | 25.01 | 34.23 | 5.46 | 7.16 | 1.42 | 0.22 | 8.65 | 0.10 | 2.78 |
| EG07 |  | 116.45 | 22.6 | 20200624 | 42 | 27.11 | 33.83 | 4.44 | 2.50 | 0.08 | 0.18 | 3.50 | 0.09 | 0.43 |
| EG08 |  | 116.55 | 22.36 | 20200624 | 18 | 29.02 | 32.23 | 6.71 | 1.91 | 0.62 | 0.29 | 0.46 | 0.11 | 0.44 |
| EG09 |  | 116.65 | 22.11 | 20200624 | 89 | 30.08 | 29.56 | 7.10 | 2.53 | 5.51 | 0.49 | 0.74 | 0.09 | 0.56 |
| EG10 |  | 116.75 | 21.86 | 20200624 | 23 | 30.47 | 28.42 | 6.45 | 1.05 | 8.70 | 0.51 | 6.12 | 0.26 | 0.51 |
| EG11 |  | 115.69 | 22.65 | 20200623 | 101 | 29.97 | 25.14 | 3.99 | 1.30 | 0.47 | 0.11 | 3.85 | 0.01 | 0.49 |
| EG12 |  | 115.79 | 22.4 | 20200623 | 33 | 30.11 | 24.29 | 7.06 | 2.36 | 0.96 | 0.42 | 2.90 | 0.08 | 0.45 |
| EG13 |  | 115.89 | 22.16 | 20200623 | 54 | 29.47 | 27.13 | 6.13 | 1.13 | 12.46 | 1.08 | 4.14 | 0.08 | 0.93 |
| EG14 |  | 115.98 | 21.92 | 20200623 | 18 | 29.04 | 31.81 | 7.09 | 1.81 | 23.74 | 1.11 | 19.64 | 0.18 | 0.57 |
| EG15 |  | 116.07 | 21.67 | 20200623 | 29 | 25.87 | 34.15 | 6.07 | 1.68 | 23.04 | 1.01 | 18.61 | 0.15 | 1.76 |
| MG01 |  | 114.99 | 22.47 | 20200621 | 11 | 28.57 | 31.75 | 7.35 | 2.59 | 0.27 | 0.18 | 4.69 | 0.05 | 1.46 |
| MG02 |  | 115.09 | 22.24 | 20200622 | 40 | 29.52 | 25.33 | 6.17 | 2.10 | 16.54 | 1.38 | 5.75 | 0.11 | 5.37 |
| MG03 |  | 115.19 | 22 | 20200622 | 11 | 30.44 | 23.09 | 6.43 | 1.92 | 18.55 | 0.91 | 15.03 | 0.24 | 0.17 |
| MG04 |  | 115.28 | 21.76 | 20200622 | 21 | 30.62 | 23.22 | 6.55 | 1.91 | 22.86 | 1.00 | 16.88 | 0.19 | 0.53 |
| MG05 |  | 115.38 | 21.51 | 20200622 | 8 | 29.95 | 34.07 | 7.32 | 1.28 | 5.00 | 0.26 | 4.98 | 0.07 | 0.07 |
| MG06 |  | 114.34 | 22.22 | 20200620 | 31 | 24.74 | 32.21 | 7.32 | 2.17 | 9.06 | 2.47 | 12.56 | 0.25 | 6.03 |
| MG07 |  | 114.44 | 21.98 | 20200620 | 48 | 34.21 | 24.61 | 4.85 | 2.82 | 25.90 | 1.42 | 18.30 | 0.25 | 0.67 |
| MG08 |  | 114.53 | 21.73 | 20200621 | 94 | 30.61 | 27.01 | 7.26 | 1.56 | 20.73 | 1.14 | 14.18 | 0.20 | 0.66 |
| MG09 |  | 114.62 | 21.48 | 20200621 | 101 | 29.45 | 34.45 | 7.41 | 1.36 | 0.18 | 0.06 | 1.96 | 0.01 | 0.10 |
| MG10 |  | 114.72 | 21.23 | 20200621 | 101 | 29.67 | 34.52 | 6.48 | 1.44 | 0.47 | 0.04 | 1.35 | 0.01 | ND |
| MG11 |  | 113.65 | 22.07 | 20200619 | 9 | 29.97 | 18.08 | 7.20 | 1.61 | 53.61 | 2.64 | 56.40 | 0.74 | 9.33 |
| MG12 |  | 113.74 | 21.82 | 20200619 | 32 | 29.57 | 16.81 | 7.18 | 2.23 | 41.47 | 1.97 | 30.53 | 0.35 | 0.45 |
| MG13 | GD | 113.84 | 21.57 | 20200619 | 44 | 30.54 | 28.89 | 7.42 | 1.80 | 7.93 | 0.55 | 6.48 | 0.20 | 0.50 |
| MG14 |  | 113.94 | 21.33 | 20200620 | 92 | 29.05 | 34.34 | 7.42 | 1.75 | 0.32 | 0.07 | 1.75 | 0.01 | 0.07 |
| MG15 |  | 114.03 | 21.07 | 20200620 | 78 | 29.27 | 33.95 | 5.21 | 1.61 | 0.38 | 0.08 | 1.60 | 0.02 | 0.13 |
| MG16 |  | 112.98 | 21.82 | 20200612 | 9 | 29.97 | 15.11 | 6.83 | 1.59 | 60.24 | 1.72 | 33.60 | 0.52 | 22.08 |
| MG17 |  | 113.08 | 21.58 | 20200618 | 33 | 30.18 | 24.48 | 7.24 | 2.84 | 12.38 | 0.89 | 6.70 | 0.21 | 2.71 |
| MG18 |  | 113.18 | 21.34 | 20200618 | 32 | 29.23 | 33.47 | 7.03 | 0.74 | 0.30 | 0.10 | 3.37 | 0.02 | 0.15 |
| MG19 |  | 113.27 | 21.09 | 20200618 | 60 | 28.32 | 33.78 | 5.62 | 1.06 | 0.43 | 0.14 | 1.20 | 0.10 | 0.01 |
| MG20 |  | 113.37 | 20.85 | 20200619 | 68 | 28.78 | 33.59 | 6.56 | 0.92 | 0.40 | 0.09 | 0.93 | 0.02 | 0.18 |
| WG01 |  | 112.42 | 21.37 | 20200611 | 33 | 29.03 | 33.10 | 7.20 | 1.28 | 0.38 | 0.10 | 1.13 | 0.11 | 0.21 |
| WG02 |  | 112.51 | 21.13 | 20200611 | 49 | 30.05 | 34.40 | 4.96 | 1.21 | 0.42 | 0.07 | 0.06 | 0.07 | ND |
| WG03 |  | 112.61 | 20.88 | 20200611 | 58 | 29.35 | 33.94 | 6.70 | 1.39 | 0.44 | 0.04 | 2.08 | 0.04 | ND |
| WG04 |  | 112.71 | 20.64 | 20200611 | 78 | 29.90 | 34.19 | 7.03 | 0.94 | 0.41 | 0.13 | 1.16 | 0.16 | ND |
| WG05 |  | 111.58 | 21.4 | 20200702 | 34 | 27.73 | 34.06 | 6.51 | 2.64 | 0.37 | 0.09 | 3.48 | 0.06 | 0.12 |
| WG06 |  | 111.71 | 21.18 | 20200703 | 36 | 30.55 | 32.79 | 3.99 | 1.75 | 0.32 | 0.06 | 1.34 | 0.04 | 0.35 |
| WG07 |  | 111.87 | 20.94 | 20200702 | 48 | 28.70 | 34.05 | 4.52 | 1.14 | 0.05 | 0.11 | 1.83 | 0.06 | ND |
| WG08 |  | 112.01 | 20.71 | 20200702 | 64 | 29.00 | 22.77 | 8.99 | 2.40 | 0.34 | 0.16 | 1.53 | 0.09 | ND |
| WG09 |  | 110.87 | 21.3 | 20200703 | 14 | 30.40 | 31.31 | 4.32 | 2.88 | 0.47 | 0.13 | 9.72 | 0.10 | 0.63 |
| WG10 |  | 111.01 | 21.06 | 20200703 | 25 | 28.71 | 33.83 | 4.58 | 1.06 | 0.67 | 0.06 | 0.83 | 0.03 | 0.30 |
| WG11 |  | 111.16 | 20.83 | 20200703 | 38 | 27.89 | 34.20 | 3.36 | 1.46 | 0.53 | 0.01 | 0.87 | 0.03 | 0.01 |
| WG12 |  | 111.3 | 20.58 | 20200703 | 52 | 27.41 | 34.53 | 4.48 | 1.36 | 0.62 | 0.09 | 2.08 | 0.01 | ND |
| BM01 | BG | 108.11 | 19.97 | 20210925 | 61 | 30.46 | 32.34 | 6.16 | 0.14 | 0.19 | 0.02 | 3.39 | 0.32 | 0.29 |
| BM02 |  | 108.31 | 19.89 | 20210925 | 62 | 30.35 | 32.56 | 6.17 | 0.18 | 0.28 | ND | 3.54 | 0.56 | 0.35 |
| BM03 |  | 108.63 | 19.75 | 20210925 | 65 | 30.12 | 32.74 | 6.14 | 0.29 | 0.23 | ND | 3.41 | 0.82 | 0.23 |
| BM04 |  | 108.86 | 19.65 | 20210809 | 35 | 29.60 | 33.78 | 6.50 | 0.20 | 0.23 | 0.02 | 3.23 | 0.58 | 0.45 |
| BM05 |  | 107.83 | 19.61 | 20210924 | 59 | 30.07 | 33.16 | 6.20 | 0.26 | 0.28 | ND | 3.15 | 0.59 | 0.21 |
| BM06 |  | 108.27 | 19.47 | 20210924 | 64 | 30.10 | 32.95 | 6.30 | 0.29 | 0.27 | ND | 3.73 | 0.26 | 0.34 |
| BM07 |  | 108.62 | 19.35 | 20210923 | 30 | 29.34 | 32.73 | 6.27 | 0.22 | 0.49 | 0.19 | 5.37 | 0.68 | 1.72 |
| BM08 |  | 107.37 | 19.19 | 20210923 | 63 | 30.41 | 33.11 | 6.21 | 0.18 | 0.23 | 0.02 | 2.55 | 0.25 | 0.13 |
| BM09 | BG | 107.64 | 19.16 | 20210923 | 63 | 30.30 | 33.07 | 6.29 | 0.14 | 0.22 | ND | 2.62 | 0.50 | 0.14 |
| BM10 |  | 108.13 | 19.12 | 20210923 | 45 | 30.15 | 33.39 | 6.26 | 0.29 | 0.23 | 0.01 | 3.05 | 0.51 | 0.15 |
| BM11 |  | 108.5 | 19.09 | 20210923 | 16 | 29.24 | 33.38 | 6.05 | 0.26 | 0.52 | 0.14 | 4.21 | 0.78 | 1.22 |
| BM12 |  | 107.49 | 18.66 | 20210922 | 61 | 29.66 | 33.53 | 6.29 | 0.18 | 0.23 | 0.01 | 2.25 | 0.62 | 0.13 |
| BM13 |  | 107.88 | 18.65 | 20210922 | 68 | 29.69 | 33.36 | 6.25 | 0.26 | 0.17 | ND | 3.16 | 0.56 | 0.20 |
| BM14 |  | 108.23 | 18.68 | 20210922 | 44 | 30.03 | 33.10 | 6.27 | 0.33 | 0.21 | 0.01 | 3.15 | 0.68 | 0.31 |
| BM15 |  | 108.5 | 18.69 | 20210922 | 23 | 29.35 | 33.45 | 6.08 | 0.29 | 0.29 | 0.09 | 2.24 | 0.59 | 0.44 |
| BN01 |  | 108.22 | 21.41 | 20210812 | 14 | 30.93 | 28.99 | 5.66 | 0.50 | 0.74 | 0.38 | 3.01 | 0.66 | 5.82 |
| BN02 |  | 108.63 | 21.5 | 20210811 | 7 | 30.71 | 28.76 | 8.97 | 0.41 | 2.86 | 0.87 | 17.80 | 0.48 | 2.42 |
| BN03 |  | 108.91 | 21.26 | 20210811 | 18 | 30.09 | 31.77 | 6.00 | 0.47 | 0.25 | 0.04 | 2.82 | 0.24 | 1.10 |
| BN04 |  | 109.25 | 21.24 | 20210810 | 18 | 30.57 | 31.31 | 6.30 | 0.32 | 0.18 | 0.06 | 3.51 | 0.25 | 1.88 |
| BN05 |  | 109.58 | 21.34 | 20210810 | 15 | 30.38 | 29.81 | 6.29 | 2.57 | 3.59 | 0.99 | 10.34 | 0.35 | 4.00 |
| BN06 |  | 108.33 | 20.94 | 20210812 | 34 | 30.78 | 31.47 | 6.65 | 0.32 | 0.17 | ND | 10.02 | 0.73 | 0.63 |
| BN07 |  | 108.58 | 21.03 | 20210811 | 33 | 30.82 | 29.48 | 6.71 | 0.23 | 0.39 | 0.01 | 7.51 | 0.45 | 0.52 |
| BN08 |  | 109.21 | 21.01 | 20210810 | 21 | 30.89 | 31.81 | 6.30 | 0.29 | 0.15 | 0.04 | 3.93 | 0.26 | 1.52 |
| BN09 |  | 109.45 | 20.88 | 20210810 | 12 | 30.69 | 32.75 | 6.18 | 0.38 | 0.18 | 0.10 | 4.77 | 0.68 | 1.34 |
| BN10 |  | 108.51 | 20.61 | 20210812 | 46 | 30.44 | 32.15 | 6.39 | 0.16 | 0.25 | 0.02 | 5.43 | 0.24 | 0.37 |
| BN11 |  | 108.82 | 20.78 | 20210811 | 34 | 30.84 | 32.02 | 6.22 | 0.26 | 0.23 | 0.03 | 4.53 | 0.21 | 0.39 |
| BN12 |  | 109.09 | 20.6 | 20210813 | 28 | 30.65 | 31.68 | 6.32 | 0.13 | 0.23 | ND | 5.61 | 0.29 | 0.31 |
| BN13 |  | 109.57 | 20.6 | 20210815 | 23 | 30.96 | 32.38 | 6.55 | 0.01 | 0.19 | 0.02 | 7.81 | 0.36 | 0.80 |
| BN14 |  | 108.41 | 20.35 | 20210925 | 60 | 30.00 | 32.57 | 6.11 | 0.26 | 0.27 | ND | 5.94 | 0.67 | 0.25 |
| BN15 |  | 108.76 | 20.33 | 20210926 | 49 | 29.44 | 32.95 | 6.17 | 0.22 | 0.21 | 0.01 | 4.50 | 0.40 | 0.31 |
| BN16 |  | 109.32 | 20.34 | 20210809 | 20 | 30.53 | 32.35 | 6.70 | 1.29 | 0.16 | 0.04 | 6.27 | 0.60 | 1.13 |
| BN17 |  | 109.64 | 20.33 | 20210815 | 14 | 30.68 | 32.17 | 6.11 | 0.07 | 0.25 | 0.12 | 7.87 | 0.41 | 0.92 |
| BS01 |  | 107.99 | 18.04 | 20210921 | 89 | 30.08 | 32.00 | 6.29 | 0.18 | 0.16 | ND | 2.75 | 0.19 | 0.23 |
| BS02 |  | 108.15 | 18.12 | 20210921 | 84 | 30.35 | 32.22 | 6.29 | 0.22 | 0.38 | ND | 3.15 | 0.24 | 0.19 |
| BS03 |  | 108.46 | 18.34 | 20210921 | 51 | 29.48 | 33.57 | 6.34 | 0.29 | 0.19 | ND | 2.88 | 0.54 | 0.13 |
| BS04 |  | 108.44 | 17.65 | 20210921 | 104 | 29.79 | 30.91 | 6.38 | 0.14 | 0.54 | ND | 2.93 | 0.54 | 0.32 |
| BS05 | BG | 108.62 | 17.9 | 20210921 | 90 | 29.81 | 31.12 | 6.26 | 0.14 | 0.53 | ND | 3.15 | 0.53 | 0.25 |
| BS06 |  | 108.77 | 18.11 | 20210920 | 48 | 29.71 | 32.68 | 6.29 | 0.32 | 0.22 | ND | 3.34 | 0.63 | 0.15 |
| BS07 |  | 109.02 | 17.24 | 20210920 | 100 | 29.46 | 33.78 | 6.31 | 0.20 | 0.22 | 0.01 | 2.25 | 0.22 | 0.08 |
| BS08 |  | 109.07 | 17.5 | 20210920 | 86 | 29.80 | 32.48 | 6.30 | 0.20 | 0.19 | ND | 3.40 | 0.51 | 0.17 |
| BS09 |  | 109.12 | 17.77 | 20210920 | 78 | 29.71 | 33.62 | 6.33 | 0.23 | 0.21 | ND | 2.59 | 0.28 | 0.13 |
| BS10 |  | 109.18 | 18.05 | 20210920 | 42 | 29.49 | 33.94 | 6.36 | 0.23 | 0.56 | 0.01 | 2.20 | 0.70 | 0.11 |
| BS11 |  | 109.64 | 17.2 | 20210919 | 150 | 28.77 | 29.05 | 6.29 | 0.29 | 0.30 | ND | 2.03 | 0.73 | 0.11 |
| BS12 |  | 109.63 | 17.51 | 20210919 | 109 | 29.74 | 33.89 | 6.29 | 0.26 | 0.25 | ND | 2.08 | 0.23 | 0.09 |
| BS13 |  | 109.64 | 17.78 | 20210919 | 96 | 29.52 | 33.03 | 6.29 | 0.29 | 0.30 | 0.01 | 1.99 | 0.52 | 0.14 |
| BS14 |  | 109.63 | 18.08 | 20210919 | 68 | 29.53 | 33.94 | 6.27 | 0.17 | 0.17 | 0.01 | 2.13 | 0.88 | 0.13 |
| QZ01 |  | 110.51 | 20.34 | 20210808 | 23 | 30.03 | 33.38 | 5.74 | 0.59 | 5.18 | 0.49 | 13.30 | 0.86 | 1.60 |
| QZ02 |  | 110.41 | 20.17 | 20210815 | 75 | 30.63 | 30.62 | 5.94 | 0.07 | 3.58 | 0.41 | 10.27 | 0.68 | 1.15 |
| QZ03 |  | 110.16 | 20.13 | 20210815 | 87 | 31.11 | 34.80 | 6.33 | 0.04 | 2.16 | 0.74 | 8.21 | 0.56 | 1.12 |
| QZ04 |  | 109.87 | 20.15 | 20210815 | 58 | 30.80 | 32.58 | 6.49 | 0.07 | 0.35 | 0.07 | 7.09 | 0.47 | 1.11 |
| QZ05 |  | 109.42 | 20.06 | 20210808 | 27 | 30.74 | 32.29 | 6.56 | 0.10 | 0.19 | 0.01 | 5.34 | 0.76 | 0.83 |
| QZ06 |  | 108.99 | 20.06 | 20210808 | 30 | 30.12 | 32.82 | 7.23 | 0.10 | 0.21 | 0.02 | 5.07 | 0.58 | 0.18 |
| WS01 | WS | 112.39 | 19.48 | 20200727 | 148 | 30.81 | 34.21 | 6.15 | ND | 1.24 | 0.02 | ND | ND | - |
| WS02 |  | 112.7 | 19.08 | 20200727 | 271 | 30.76 | 34.09 | 6.16 | 0.54 | 10.65 | ND | 5.10 | ND | - |
| WS03 |  | 113 | 18.67 | 20200727 | 1184 | 30.77 | 34.22 | 6.16 | 0.06 | 1.46 | ND | 0.14 | 0.01 | 0.12 |
| WS04 |  | 114 | 18.67 | 20200726 | 1663 | 30.77 | 34.03 | 6.16 | 0.28 | 3.45 | 0.05 | 1.31 | 0.01 | 0.02 |
| WS05 |  | 115 | 18.67 | 20200726 | 3633 | 31.63 | 34.03 | 6.08 | 0.29 | 19.02 | 0.01 | 7.56 | ND | 0.12 |
| WS06 |  | 116 | 18.67 | 20200726 | 3620 | 31.17 | 34.23 | 6.12 | 0.02 | 1.96 | ND | 0.33 | 0.05 | 0.02 |
| WS07 |  | 112.19 | 18.09 | 20200728 | 2409 | 29.98 | 34.00 | 6.24 | 0.15 | 8.08 | ND | 3.21 | ND | 0.12 |
| WS08 |  | 113 | 17.67 | 20200728 | 1618 | 30.44 | 34.10 | 6.19 | 0.10 | 1.06 | 0.04 | 0.40 | ND | 0.12 |
| WS09 |  | 114 | 17.67 | 20200728 | 3458 | 31.36 | 34.02 | 6.11 | 0.09 | 0.78 | ND | 0.30 | ND | 0.10 |
| WS10 |  | 115 | 17.67 | 20200729 | 3715 | 31.65 | 34.22 | 6.07 | 0.16 | 1.21 | 0.03 | ND | ND | ND |
| WS11 |  | 116 | 17.67 | 20200729 | 3895 | 31.23 | 34.07 | 6.12 | 0.30 | 18.04 | 0.05 | 7.33 | 0.01 | 0.02 |
| WS12 |  | 111.41 | 17.48 | 20200805 | 1429 | 30.26 | 33.98 | 6.22 | 0.10 | 0.49 | ND | ND | ND | 0.12 |
| WS13 | WS | 112 | 16.67 | 20200730 | 896 | 30.63 | 33.85 | 6.18 | 0.22 | 11.65 | 0.01 | 8.44 | ND | 0.22 |
| WS14 |  | 113 | 16.67 | 20200730 | 1463 | 30.79 | 33.89 | 6.17 | 0.76 | 0.54 | 0.05 | 0.53 | ND | 0.12 |
| WS15 |  | 114 | 16.67 | 20200730 | 3233 | 31.21 | 34.20 | 6.11 | 0.55 | 0.67 | ND | 0.63 | ND | 0.10 |
| WS16 |  | 115 | 16.67 | 20200729 | 2159 | 31.31 | 34.13 | 6.11 | 0.62 | 21.69 | 0.01 | 6.94 | ND | 0.10 |
| WS17 |  | 116 | 16.67 | 20200729 | 4066 | 31.34 | 34.04 | 6.11 | 0.55 | 0.06 | 0.05 | ND | ND | 0.12 |
| WS18 |  | 111 | 17.04 | 20200809 | 1434 | 30.58 | 33.74 | 6.19 | 0.08 | 1.37 | 0.03 | 1.77 | 0.01 | 0.12 |
| WS19 |  | 111.67 | 16.08 | 20200808 | 1209 | 30.48 | 34.07 | 6.19 | 0.68 | 1.72 | ND | 0.99 | ND | 0.02 |
| WS20 |  | 112 | 15.67 | 20200808 | 1313 | 30.24 | 34.04 | 6.22 | 0.06 | 0.84 | 0.04 | ND | 0.01 | 0.02 |
| WS21 |  | 113 | 15.67 | 20200807 | 2614 | 30.06 | 33.94 | 6.24 | 0.06 | 2.26 | 0.03 | 1.64 | 0.03 | 0.02 |
| WS22 |  | 113.6 | 15.67 | 20200807 | 2020 | 30.02 | 33.72 | 6.25 | 0.20 | 0.47 | ND | 0.17 | ND | 0.12 |
| WS23 |  | 115 | 15.67 | 20200806 | 4194 | 30.39 | 34.10 | 6.20 | 0.07 | 0.34 | ND | 0.53 | ND | 0.12 |
| WS24 |  | 116 | 15.67 | 20200806 | 3963 | 30.04 | 34.04 | 6.24 | 0.25 | 0.84 | 0.01 | 1.87 | ND | 0.09 |
| EH01 |  | 111.1 | 20 | 20200601 | 47 | 25.63 | 33.25 | 7.36 | 1.53 | 1.03 | 0.42 | 2.94 | 0.08 | 1.01 |
| EH02 |  | 111.47 | 20 | 20200601 | 71 | 29.90 | 36.92 | 7.26 | 1.41 | 0.17 | 0.05 | 0.07 | 0.02 | 0.42 |
| EH03 |  | 111.65 | 20 | 20200601 | 83 | 28.44 | 33.71 | 7.26 | 1.45 | 0.36 | 0.03 | 2.62 | 0.04 | 0.04 |
| EH04 |  | 112.03 | 20 | 20200601 | 101 | 29.21 | 33.05 | 6.88 | 3.09 | 0.39 | 0.01 | 1.82 | 0.01 | 0.15 |
| EH05 |  | 110.9 | 19.41 | 20200603 | 40 | 26.00 | 34.42 | 7.32 | 0.91 | 0.23 | 0.17 | 3.55 | 0.07 | 0.05 |
| EH06 |  | 111.25 | 19.2 | 20200603 | 101 | 28.75 | 34.29 | 7.11 | 2.04 | 0.33 | 0.11 | 2.37 | 0.14 | 0.04 |
| EH07 |  | 111.58 | 18.93 | 20200603 | 158 | 30.96 | 34.14 | 6.14 | ND | 0.36 | 0.03 | 0.63 | ND | 0.01 |
| EH08 |  | 110.7 | 18.91 | 20200604 | 46 | 30.26 | 34.28 | 7.19 | 1.79 | 0.32 | 0.05 | 1.40 | ND | 0.01 |
| EH09 |  | 110.9 | 18.7 | 20200604 | 50 | 29.97 | 28.35 | 5.79 | 1.46 | 0.33 | 0.12 | 0.89 | 0.15 | ND |
| EH10 |  | 111.1 | 18.5 | 20200608 | 84 | 26.54 | 34.25 | 6.04 | 1.48 | 0.43 | 0.06 | 1.44 | 0.08 | 0.04 |
| EH11 |  | 111.3 | 18.3 | 20200608 | 47 | 28.21 | 34.24 | 7.08 | 1.23 | 0.36 | 0.12 | 2.14 | 0.08 | 0.01 |
| EH12 |  | 110.27 | 18.37 | 20200609 | 91 | 30.79 | 34.31 | 7.30 | 1.00 | 0.44 | 0.07 | 1.49 | 0.05 | ND |
| EH13 |  | 110.42 | 18.13 | 20200609 | 59 | 30.67 | 35.11 | 7.15 | 3.14 | 0.40 | 0.10 | 2.14 | 0.06 | ND |
| EH14 |  | 110.58 | 17.87 | 20200609 | 104 | 30.77 | 34.35 | 6.87 | 2.19 | 0.46 | 0.02 | 1.54 | 0.04 | 0.01 |
| EH15 |  | 110.73 | 17.62 | 20200609 | 90 | 30.65 | 39.23 | 7.28 | 2.23 | 0.35 | 0.11 | 1.61 | 0.03 | 0.01 |

ND: No detected data, -: Missing data
